# Host-associated mitochondrial structure and broad geographic connectivity in Drepanocephalus associated with migratory cormorants

**DOI:** 10.1016/j.ijppaw.2026.101290

**Published:** 2026-09-18

**Authors:** Daniel Muñoz, Stephen E. Harris, Erika T. Ebbs

**Affiliations:** Purchase College, State University of New York, 735 Anderson Hill Rd, Purchase, NY, 10577, USA

**Keywords:** *Drepanocephalus*, Echinostomatidae, Mitogenomics, Wildlife parasitology, Aquaculture, Migratory birds

## Abstract

Digenetic trematodes are important parasites of aquaculture systems, where transmission may be amplified by interactions among aquatic birds, fishes, and snail intermediate hosts. Species boundaries and transmission dynamics within the widespread echinostomatid genus *Drepanocephalus* remain poorly understood despite reports of pathogenicity in North American aquaculture facilities. Here, we characterize *Drepanocephalus* recovered from Double-crested Cormorants (*Nannopterum auritus*) in New York State and evaluate their evolutionary relationships using multilocus phylogenetic analyses and next-generation sequencing. Fifteen cormorants were examined, of which seven (47%) were infected with *Drepanocephalus*. Long-read and short-read sequencing recovered the complete rRNA operon and a near-complete mitochondrial genome, providing the first mitochondrial genomic resources for the genus. Phylogenetic analyses of mitochondrial (*cox*1, *nad*1) and nuclear (28S, ITS1–5.8S–ITS2) markers recovered shallow but persistent mitochondrial structure within the *D. spathans/auritus* complex. Mitochondrial loci and GMYC analyses consistently recovered two major intraspecific lineages, whereas nuclear markers recovered substantially weaker divergence and limited phylogenetic resolution, suggesting incomplete lineage sorting, ongoing gene flow, or both. Haplotype analyses further indicated that mitochondrial structure was more strongly associated with host use than with geographic locality, with lineages largely partitioned between Double-crested and Neotropic cormorant (*N. brasilianus*) transmission systems. Together, these results support broad parasite connectivity across the Americas while revealing previously undescribed host-associated mitochondrial structure. Our findings provide foundational genomic resources for future studies and highlight the role of host ecology in shaping parasite diversification and transmission across continental scales.

## Highlights

• Three nearly-complete mitogenomes generated for *Drepanocephalus*.
• Multilocus data reveal shallow divergence within the *D.* cf. *spathans* complex.
• Two mitochondrial lineages occur across the Americas associated with host use.
• Host ecology may structure parasite lineages across transmission systems.
• Broad parasite connectivity persists despite mitochondrial structure.

## Graphical abstract

**Figure:**
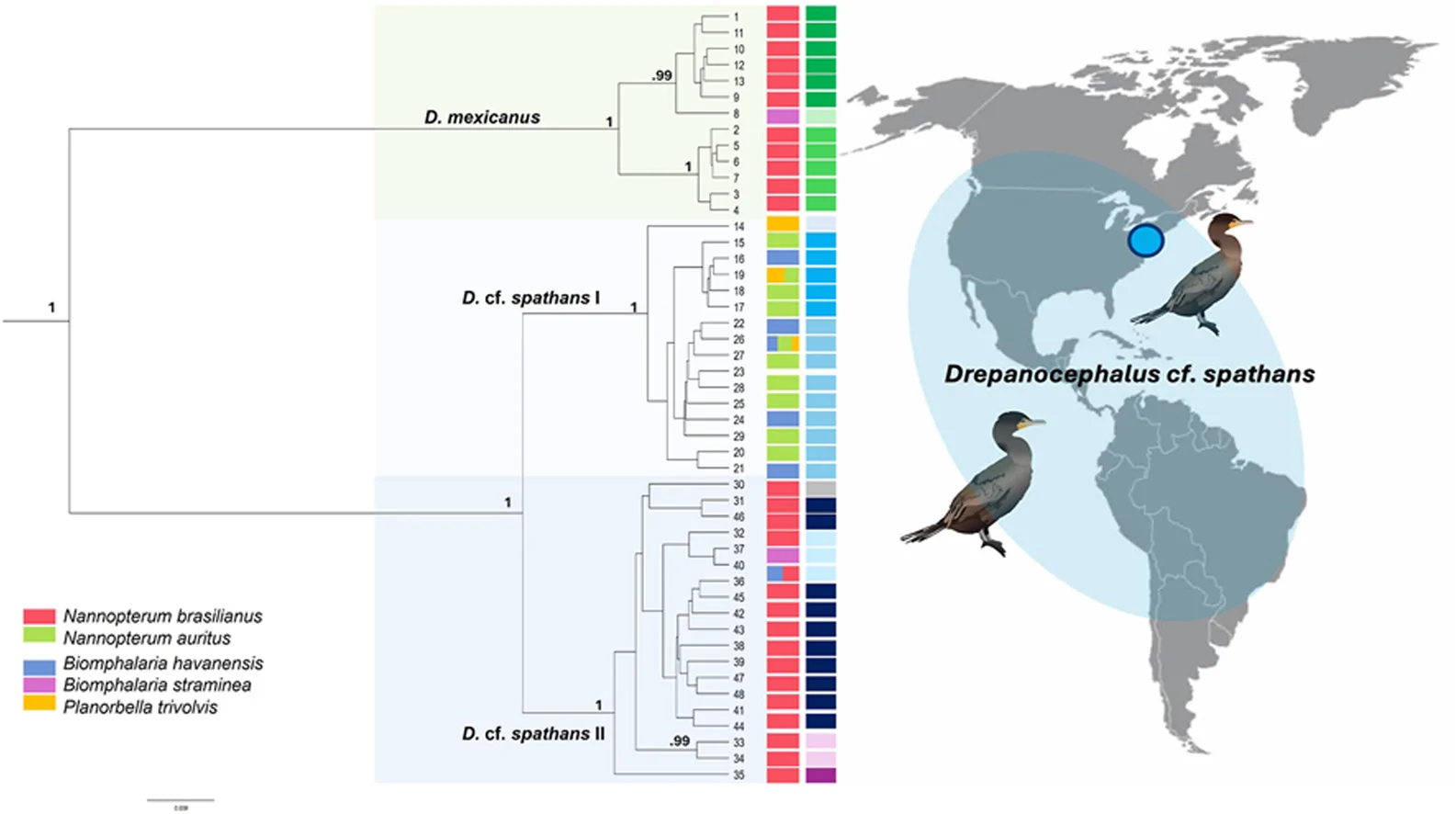

## 1 Introduction

Despite contributing substantially to global animal protein production, aquaculture facilities are frequently impacted by parasitic disease outbreaks that result in significant economic losses (FAO, 2020). Among these parasites, digenetic trematodes are of particular concern because of their complex life cycles involving aquatic intermediate hosts and piscivorous birds (Madsen et al., 2022). Within aquaculture systems, transmission typically involves a snail first intermediate host, a fish or other aquatic vertebrate second intermediate host, and an avian definitive host, all of which may occur at high densities (Roberts et al., 1996; Griffin et al., 2012; Madsen et al., 2022). Consequently, aquaculture facilities can serve as ecological interfaces that facilitate and amplify parasite transmission (Paladini et al., 2017; Madsen et al., 2022). Understanding the diversity, transmission dynamics, and geographic distribution of trematode parasites is therefore critical for predicting outbreaks and managing economically important host–parasite systems (Griffin et al., 2012, 2014).

Several digenean trematodes associated with aquaculture belong to the family Echinostomatidae Paladini et al. (2017). Echinostomatids are cosmopolitan parasites capable of infecting a broad range of definitive hosts, including birds and mammals (Detwiler et al., 2010). One echinostomatid genus commonly reported from North American aquaculture systems is *Drepanocephalus* (Griffin et al. (2012). Adult *Drepanocephalus* infect piscivorous birds, particularly the Neotropic Cormorant (*Nannopterum brasilianum*) and Double-crested Cormorant (*Nannopterum auritus*) (Hernández-Cruz et al., 2017).

These host species differ markedly in their geographic distributions and movement ecology (Kennedy and Spencer, 2014; Harrell, 2016). Double-crested cormorants are highly migratory, breeding throughout much of the United States and southern Canada and moving along continental flyways between breeding and wintering grounds (King et al., 2010). In contrast, Neotropic cormorants are largely resident throughout Central and South America, with the northern extent of their range overlapping that of Double-crested Cormorants in Mexico and the southeastern United States (Harrell, 2016). Previous work has demonstrated that parasite assemblages differ between migratory and resident cormorant populations (Sheehan et al., 2017), suggesting that host movement ecology may influence parasite transmission dynamics, population connectivity, and phylogeographic structure.

Reports of *Drepanocephalus* from first intermediate hosts remain relatively limited, but available records demonstrate that several members of the family Planorbidae participate in transmission, including *Biomphalaria straminea*, *B. havanensis*, and *Planorbella trivolvis* (Pinto et al., 2016; Alberson et al., 2017; McPhail et al., 2025). Among these, *B. havanensis* has been repeatedly implicated in transmission within aquaculture systems in the southeastern United States (Griffin et al., 2012; Alberson et al., 2017), whereas *P. trivolvis* has been shown to host *Drepanocephalus* cf. *spathans* under both natural and experimental conditions (Gordy and Hanington, 2019; Alberson et al., 2022).

These snail hosts differ markedly in their geographic distributions. *Biomphalaria straminea* is widespread throughout tropical and subtropical regions of South America, whereas *B. havanensis* occurs throughout the Caribbean, Mexico, and the southeastern United States (Yong et al., 2001; Thompson, 2011; Alberson et al., 2017). In contrast, *Planorbella trivolvis* is broadly distributed across temperate North America, extending from Canada to Mexico (Cózatl-Manzano and Naranjo-García, 2007; Gordy and Hanington, 2019; Martin et al., 2020). The broad distributions of these planorbid hosts suggest that suitable intermediate hosts for *Drepanocephalus* transmission occur across much of the parasite's known range and may contribute to patterns of parasite connectivity and phylogeographic structure.

Even more scant are reports of *Drepanocephalus* from second intermediate hosts outside of the aquaculture setting. Metacercariae of *Drepanocephalus* have been reported from several freshwater fish species, including economically important hosts such as the Channel Catfish (*Ictalurus punctatus*) (Griffin et al., 2012). In aquaculture systems, these parasites encyst within the cranial tissues and branchial chambers of juvenile catfish, resulting in tissue damage, impaired respiration, and, in severe cases, mortality (Griffin et al., 2012, 2014). Consequently, *Drepanocephalus* has emerged as an economically important parasite of North American aquaculture.

Molecular studies have also suggested broad geographic connectivity among *Drepanocephalus* populations. Alberson et al. (2017) demonstrated that parasites transmitted within aquaculture ponds in the southeastern United States were genetically similar to populations reported from Mexico and South America. These findings suggest either extensive parasite dispersal across the Americas or unresolved species boundaries within the genus.

Accurate species identification is essential for understanding the transmission ecology, host specificity, and geographic distribution of trematode pathogens within aquaculture systems. However, species boundaries within *Drepanocephalus* remain unresolved, particularly among North and South American populations, which have historically been identified as *D. spathans*. Kudlai et al. (2015) examined specimens from multiple localities in the United States and reported morphological differences relative to *D. spathans* from Mexico and South America. Based on these observations, they described *Drepanocephalus auritus* from Double-crested Cormorants (*Nannopterum auritus*), proposing that North American populations represented a distinct species.

Increased geographic and molecular sampling subsequently challenged this interpretation. Pinto et al. (2016) and Hernández-Cruz et al. (2017) recovered shallow genetic divergence among North and South American populations, and Hernández-Cruz et al. (2017) treated *D. auritus* as a junior synonym of *D. spathans*. Alberson et al. (2022) further demonstrated substantial morphological plasticity among experimentally and naturally infected specimens, although their study did not explicitly compare the mitochondrial lineages identified here. Thus, *D. auritus* has generally been treated as synonymous with *D. spathans* following Hernández-Cruz et al. (2017). However, expanded geographic and genetic sampling provides an opportunity to reevaluate lineage structure within this broadly distributed taxon.

Mitochondrial genomes have become valuable resources for resolving phylogenetic relationships, assessing population structure, and examining evolutionary divergence among closely related parasite taxa (Littlewood, 2006; Brabec et al., 2015). Compared with many nuclear markers, mitochondrial loci typically exhibit elevated substitution rates, limited recombination, and high copy number, making them particularly useful for detecting recent evolutionary divergence and cryptic diversity (Avise, 2000; Brabec et al., 2015). Despite their utility, genomic resources for *Drepanocephalus* remain limited, and no complete mitochondrial genome has been reported for the genus. Mitogenomic data have also proven informative for resolving phylogenetic relationships in other echinostomatids (Li and Durbin, 2009; Gacad et al., 2023), including the closely related genus *Chaunocephalus* (Zhou et al., 2025), suggesting that similar approaches may help clarify species boundaries and phylogeographic patterns within *Drepanocephalus*. Consequently, generating mitochondrial genomic resources represents an important step toward understanding the evolutionary history, geographic structure, and taxonomic status of this economically important parasite group.

The present study integrates mitochondrial genomics, multilocus phylogenetics, and haplotype network analyses to investigate species boundaries and phylogeographic structure within *Drepanocephalus*. Specifically, we evaluate whether populations recovered from Double-crested Cormorants (*Nannopterum auritus*) in the northeastern United States represent a distinct lineage or form part of a geographically widespread transmission system extending across the Americas. By expanding geographic sampling and generating the first near-complete mitochondrial genomes for Drepanocephalus, this study provides new insights into species boundaries, host-associated population structure, and parasite connectivity across continental transmission systems.

## 2 Materials & methods

### 2.1 Host collection and parasite recovery

The Double-crested Cormorant (*Nannopterum auritus*) is considered a nuisance species in portions of New York State, where population management is periodically conducted by the New York State Department of Environmental Conservation (NYSDEC). In 2022, a cohort of *N. auritus* culled from a breeding colony on the Niagara River near Buffalo, New York, USA, was donated for parasitological examination. Hosts were stored frozen (−20 °C) until 24 h prior to necropsy, when they were thawed at 4 °C. A total of 15 hosts were necropsied for this study.

Necropsies were conducted following standard parasitological procedures (Pritchard and Kruse, 1982). The gastrointestinal tract, liver, heart, and associated viscera were examined for helminth parasites. Recovered helminths were rinsed in deionized water and preserved in either 70% or 95% ethanol for subsequent morphological and molecular analyses. In addition, respiratory and cloacal swabs, stomach contents, and fecal samples were collected and preserved for future investigations.

All host and parasite specimens were accessioned into the Museum of Southwestern Biology, Division of Parasites (MSB:Para:54101–54129), and are publicly accessible through Arctos (https://arctos.database.museum). Helminths were examined using stereoscopic and compound light microscopy and identified to the lowest possible taxonomic level using published taxonomic keys. Large echinostomatid trematodes recovered from the intestine were tentatively identified as *Drepanocephalus* sp. based on gross morphology and preserved in 95% ethanol at −20 °C for downstream molecular analyses. Representative specimens were selected from multiple hosts for molecular analyses to capture variation in infection intensity and parasite morphology.

### 2.2 DNA extraction

Fifteen adult *Drepanocephalus*-like specimens recovered from *Nannopterum auritus* were selected for molecular analyses. To maximize the likelihood of recovering mitochondrial DNA, specimens were preferentially selected from infrapopulations with the highest infection intensities. Genomic DNA was extracted from whole individual trematodes using the QIAamp DNA Micro Kit (Qiagen, Hilden, Germany) following the manufacturer's protocol for animal tissues. DNA was eluted in 60 μL of AE buffer and stored at −20 °C until further analysis.

DNA quantity and purity were assessed using a NanoDrop spectrophotometer (Thermo Fisher Scientific, Waltham, MA, USA) and a Qubit fluorometer (Thermo Fisher Scientific) prior to downstream molecular analyses.

### 2.3 Oxford nanopore sequencing

Sequencing libraries were prepared from genomic DNA of eight *Drepanocephalus*-like specimens using the Rapid Barcoding Kit v14 (SQK-RBK114.24; Oxford Nanopore Technologies, Oxford, UK) following the manufacturer's protocol. Libraries were pooled and sequenced on a FLO-MIN114 flow cell using MinKNOW v21.05.12 (Oxford Nanopore Technologies).

Raw signal data were basecalled and demultiplexed using Guppy v5.0.12 (Oxford Nanopore Technologies). Reads were quality filtered with NanoFilt v2.8.0, retaining reads with a minimum Phred quality score of Q10. To reduce contamination, filtered reads were mapped against reference genomes representing common contaminants (avian host, human, and bacterial genomes) using Minimap2 v2.22 (Li, 2018), and unmapped reads were retained for downstream analyses.

Filtered trematode reads were imported into Geneious Prime v2026.1.1 (Biomatters Ltd., Auckland, New Zealand) and mapped to the mitochondrial genome of *Chaunocephalus ferox* (GenBank accession PV685907) and the nuclear rRNA operon of *Drepanocephalus auritus* (GenBank accession KP053260). Reference-guided consensus sequences were generated from mapped reads and incorporated into locus-specific alignments for subsequent phylogenetic analyses.

### 2.4 Illumina sequencing

Three *Drepanocephalus* specimens were submitted to Novogene (Sacramento, California, USA) for whole-genome Illumina sequencing. Genomic DNA was extracted from individual adult specimens as described above. DNA quality and concentration were assessed prior to library preparation. Sequencing libraries were generated and sequenced on an Illumina NovaSeq X Plus platform using paired-end 150-bp chemistry, generating approximately 12 Gb of raw sequence data per sample.

Raw reads were quality filtered by Novogene to remove adapter contamination and low-quality sequences prior to downstream analyses. Mitochondrial genome assemblies were generated using GetOrganelle v1.7.6 (Jin et al., 2020). Resulting contigs were assessed for completeness and annotated using MITOS2 (Bernt et al., 2013), implemented through the Galaxy platform (The Galaxy Community, 2024), specifying the trematode mitochondrial genetic code and metazoan reference database. Gene annotations were manually inspected and refined in Geneious Prime v2026.1.1 (Biomatters Ltd., Auckland, New Zealand) through comparison with published echinostomatid mitochondrial genomes (e.g., Li and Durbin, 2009; Gacad et al., 2023).

To evaluate assembly quality, cleaned reads were mapped back to assembled mitochondrial contigs using BWA-MEM v0.7.17 (Li and Durbin, 2009), and alignment statistics were calculated using SAMtools v1.22 (Danecek et al., 2021). Genome completeness was assessed based on recovery of the expected trematode mitochondrial gene complement, read-mapping coverage, and assembly continuity. Assemblies were considered near-complete when all expected protein-coding genes, tRNAs, and mitochondrial rRNA genes were recovered.

Partial nuclear rRNA operons were reconstructed by reference-guided assembly using the published *Drepanocephalus auritus* rRNA operon (GenBank accession KP053260). Filtered reads were mapped using BWA-MEM v0.7.17 (Li and Durbin, 2009), and alignment files were processed with SAMtools v1.22 (Danecek et al., 2021) for sorting, indexing, and coverage estimation. Consensus sequences were generated using BCFtools v1.22 (Danecek et al., 2021) and incorporated into locus-specific alignments for subsequent phylogenetic analyses. Sequence depth and coverage statistics were calculated from mapped reads using SAMtools.

### 2.5 Phylogenetic analyses

All alignments were generated in Geneious Prime using CLUSTALW (Larkin et al., 2007). Alignments were manually inspected and trimmed to preserve reading frame integrity and remove ambiguous terminal regions. For each gene, all published *Drepanocephalus* sequences were included. Additional echinostomatid and outgroup taxa were selected based on the phylogenetic framework of Hernández-Cruz et al. (2017).

Maximum likelihood (ML) analyses were performed using RAxML v8.2.12 (Stamatakis, 2014) within Geneious Prime with 1000 bootstrap replicates under a GTR + GAMMA substitution model. Bayesian inference (BI) analyses were conducted in BEAST v2.5 (Bouckaert et al., 2019). Models of nucleotide substitution were selected using jModelTest v2.1.10 (Posada, 2008) under the Akaike Information Criterion (AIC) and implemented in Bayesian analyses. Broad phylogenetic datasets including outgroup taxa were analyzed under a Yule speciation prior, whereas analyses restricted to *Drepanocephalus* haplotypes were performed under a Constant Population Coalescent prior. All analyses employed a strict molecular clock and were run for 10,000,000 generations, sampling every 1000 generations. Convergence and parameter mixing were assessed in Tracer v1.6 (Rambaut et al., 2014), and all reported analyses achieved ESS values > 200. Maximum clade credibility trees were generated following a 10% burn-in and visualized using FigTree v1.4.4 (Rambaut, 2018).

Individual gene tree datasets consisted of *cox*1 (n = 98, 537 bp), *nad*1 (n = 66, 429 bp), 28S (n = 48, 1025 bp), and ITS1+5.8S + ITS2 (n = 96, 1010 bp). Species boundaries within *Drepanocephalus* were assessed using a Generalized Mixed Yule Coalescent (GMYC) approach (Fujisawa and Barraclough, 2013). An ultrametric *cox*1 tree comprising *Drepanocephalus* sequences (n = 83, 537 bp) was generated in BEAST v2.5 under a Constant Population Coalescent prior and an HKY + GAMMA substitution model with four gamma categories and a chain length of 5,000,000 generations. Posterior distributions were evaluated in Tracer v1.6, and all parameters achieved ESS values > 200. A maximum clade credibility ultrametric tree was generated following a 10% burn-in. The resulting ultrametric tree was analyzed using the R package splits (Fujisawa and Barraclough, 2013) to perform a single-threshold GMYC species delimitation.

### 2.6 Genetic distance and haplotype analyses

The *Drepanocephalus*-only *cox*1 alignment used for GMYC analyses was additionally used to calculate population genetic summary statistics in DnaSP v6 (Rozas et al., 2017), including the number of segregating sites (S), nucleotide diversity (π), haplotype diversity (Hd), Watterson's theta (θ_W_), and Tajima's D. Sequences were subsequently collapsed into unique haplotypes, resulting in a dataset of 48 haplotypes comprising 123 variable sites. Relationships among haplotypes were visualized using minimum spanning networks (MSNs; Bandelt et al., 1999) constructed in PopArt v1.7 (Leigh and Bryant, 2015). Separate networks were generated to examine patterns associated with host taxon, geographic locality, and GMYC cluster assignment.

To further evaluate relationships among unique haplotypes, Bayesian phylogenetic analyses were conducted in BEAST v2.5 (Bouckaert et al., 2019) under a strict molecular clock and Constant Population Coalescent prior. Analyses were run for 10,000,000 generations, sampling every 1000 generations. Maximum clade credibility trees were generated following a 10% burn-in and visualized in FigTree v1.4.4 (Rambaut, 2018). Pairwise uncorrected p-distances were calculated in Geneious Prime to quantify genetic divergence within and among mitochondrial lineages and *Drepanocephalus* species.

## 3 Results

### 3.1 Host infection patterns

A total of 15 Double-crested Cormorants (*Nannopterum auritus*) were necropsied and examined for helminth parasites (Supplementary File 1). Overall, 93% of hosts were infected with at least one helminth species. *Drepanocephalus* (Digenea: Echinostomatidae) was the most frequently recovered taxon, occurring at a prevalence of 47% and a mean intensity of 10.5 worms per infected host. Specimens from three infected hosts (HP1010, HP1183, and HP1189) were selected for genomic sequencing and subsequent phylogenetic analyses. All published reports of *Drepanocephalus* spp. were assembled in Supplementary File 2.

### 3.2 Next-generation sequencing

#### 3.2.1 MinION ONT sequencing summary

Genomic DNA from 8 individual *Drepanocephalus* specimens was prepared for ONT long-read sequencing. Overall, coverage of phylogenetically informative markers was low. No complete or near-complete mitochondrial genomes were recovered, and all samples lacked sufficient c*ox*1 coverage for reliable contig assembly. However, most of the rRNA operon was recovered from all specimens and assembled into useable consensus sequences (Supplementary File 3). Consequently, ONT data were used primarily to recover rRNA markers, whereas mitochondrial analyses were based on Illumina sequencing. Across samples, the rRNA operon had a mean sequencing depth of 4.18×, with an average of 132.67 reads mapping to the 4127-bp reference sequence.

#### 3.2.2 Illumina sequencing summary

Three *Drepanocephalus* specimens were submitted for whole-genome Illumina sequencing (Table 1). For all samples, a near-complete mitochondrial genome (13,818 bp; mean depth = 744.6×) and partial rRNA operons (4267 bp; mean depth = 32,786.7×) were recovered. Mitochondrial genome coverage and sequencing depth were assessed by mapping cleaned Illumina reads back to the assembled mitochondrial contigs (Supplementary Files 4 and 5).

**Table 1:** Summary of phylogenetically relevant Illumina data, mito = mitochondrial genome assemblies; rrna = complete rRNA operon (18S, ITS1, 5s.8S, ITS2, 28S). 'Reads' refers to the number of cleaned reads that mapped back to the mitochondrial contig produced by GetOrganelle. 'Avg. Depth' refers to the average number of reads per site for the mitochondrial contig. rRNA coverage is based on 4267 bp reference of *Drepanocephalus auritus* (KP053260).

Sample	Source	Bases	Reads	Coverage	Avg. Depth
HP1183.1A	Mitochondrial	13,818	44,051	100	462
	rRNA	4267	814,434	100	27,703
HP1183.1B	Mitochondrial	13,818	69,676	100	735
	rRNA	4267	1,025,003	100	34,887
HP1189.2	Mitochondrial	13,818	124,257	100	1037
	rRNA	4267	1,051,778	100	35,770

Two specimens (HP1183.1A and HP1183.1B) were recovered from the same host individual, representing a shared infrapopulation, whereas HP1189.2 was recovered from a different host within the same colony, representing the component population. BLAST searches of partial *cox*1 sequences recovered the highest similarity to published *Drepanocephalus spathans* and *D. auritus* sequences.

#### 3.2.3 Mitochondrial genome assemblies

MITOS annotation recovered the expected trematode mitochondrial gene complement in all three assemblies (13,818 bp; Supplementary Files 4 and 5). Although gene content was complete, none of the assemblies fully circularized, suggesting incomplete recovery of the mitochondrial control region. Each assembly contained 12 protein-coding genes, 22 tRNAs, and 2 ribosomal RNA genes, consistent with published echinostomatid mitochondrial genomes (Supplementary File 6). As reported for other trematodes (Zhou et al., 2025), the mitochondrial genomes lacked the ATP synthase subunit 8 (atp8) gene and exhibited an overlap between the 3′ end of nad4L and the 5′ end of nad4. The GC content was identical among all three assemblies (37.4%).

Comparisons among mitochondrial genomes recovered from different hosts identified relatively low levels of sequence variation. Commonly used phylogenetic markers (*cox*1and *nad*1) contained comparatively few polymorphic sites, whereas genes such as cytb and *nad*5exhibited greater sequence variation among samples (Supplementary File 5). These results suggest that additional mitochondrial loci may provide greater resolution for future population genetic and phylogeographic studies of *Drepanocephalus*.

### 3.3 Phylogenetic relationships among Drepanocephalus spp.

All gene trees (Fig. 1, Fig. 2, Fig. 3, Supplementary File 7) were congruent between Maximum Likelihood (ML) and Bayesian Inference (BI) analyses in supporting the monophyly of *Drepanocephalus*, including sequences designated as *D. spathans*, *D. auritus*, and *D. mexicanus* (all gene trees: bs = 100, pp = 1). *Chaunocephalus ferox* was consistently recovered as the sister taxon to *Drepanocephalus* in mitochondrial and ITS phylogenies (ITS region: bs = 97; *cox*1: bs = 51), with the exception of the 28S phylogeny, in which it was recovered as sister to the *Drepanocephalus* + *Petasiger* clade (bs = 100; Supplementary File 7).

**Fig. 1:**
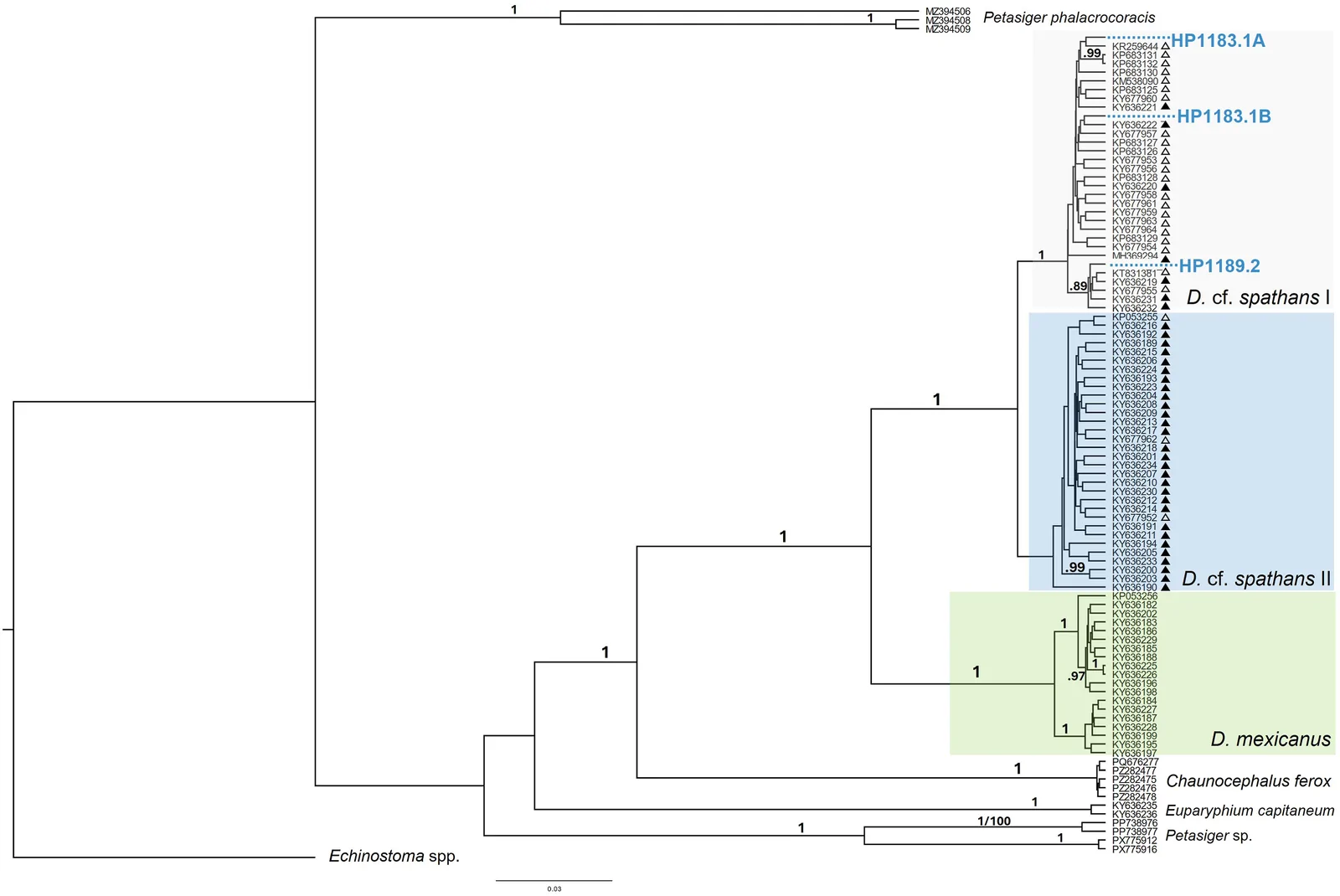
Bayesian phylogeny inferred from *cox*1 sequences using BEAST v2.5 and visualized in FigTree. Posterior probabilities ≥ 0.89 are indicated at the corresponding nodes. The two mitochondrial clades within the *D. spathans*/*auritus* complex are designated *D.* cf. *spathans* I and *D.* cf. *spathans* II. Solid triangles indicate sequences designated as *D. spathans,* open triangles indicate sequences designated as *D. auritus*.

**Fig. 2:**
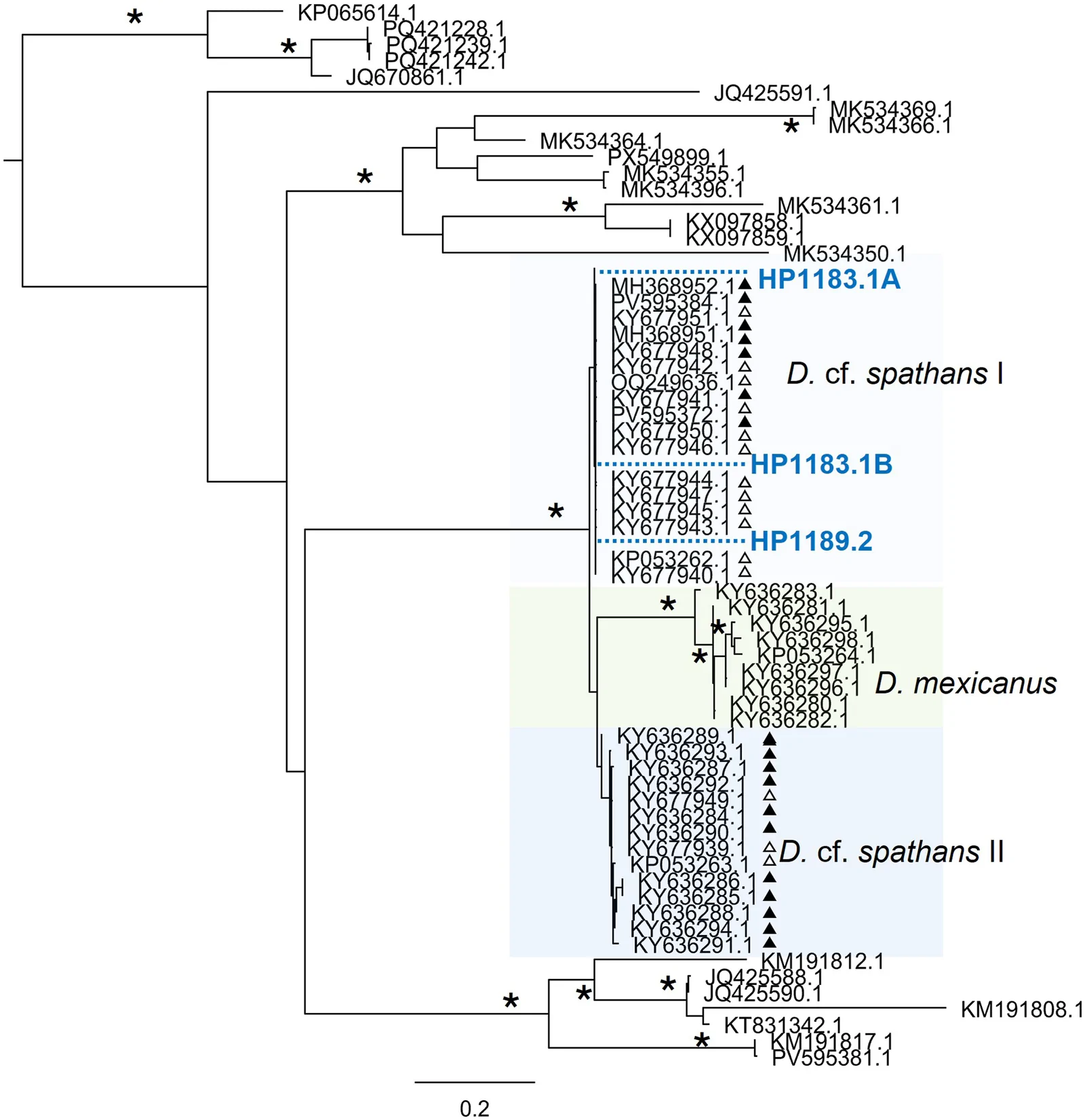
Maximum likelihood (ML) phylogeny inferred from *nad*1 sequences. Bootstrap support values ≥ 70 are indicated by an asterisk (*); exact bootstrap values are reported in the Results. Two mitochondrial clades within the *D. spathans*/*auritus* complex were recovered and are designated *D.* cf. *spathans* I and *D.* cf. *spathans* II. Solid triangles indicate sequences designated as *D. spathans,* open triangles indicate sequences designated as *D. auritus*.

**Fig. 3:**
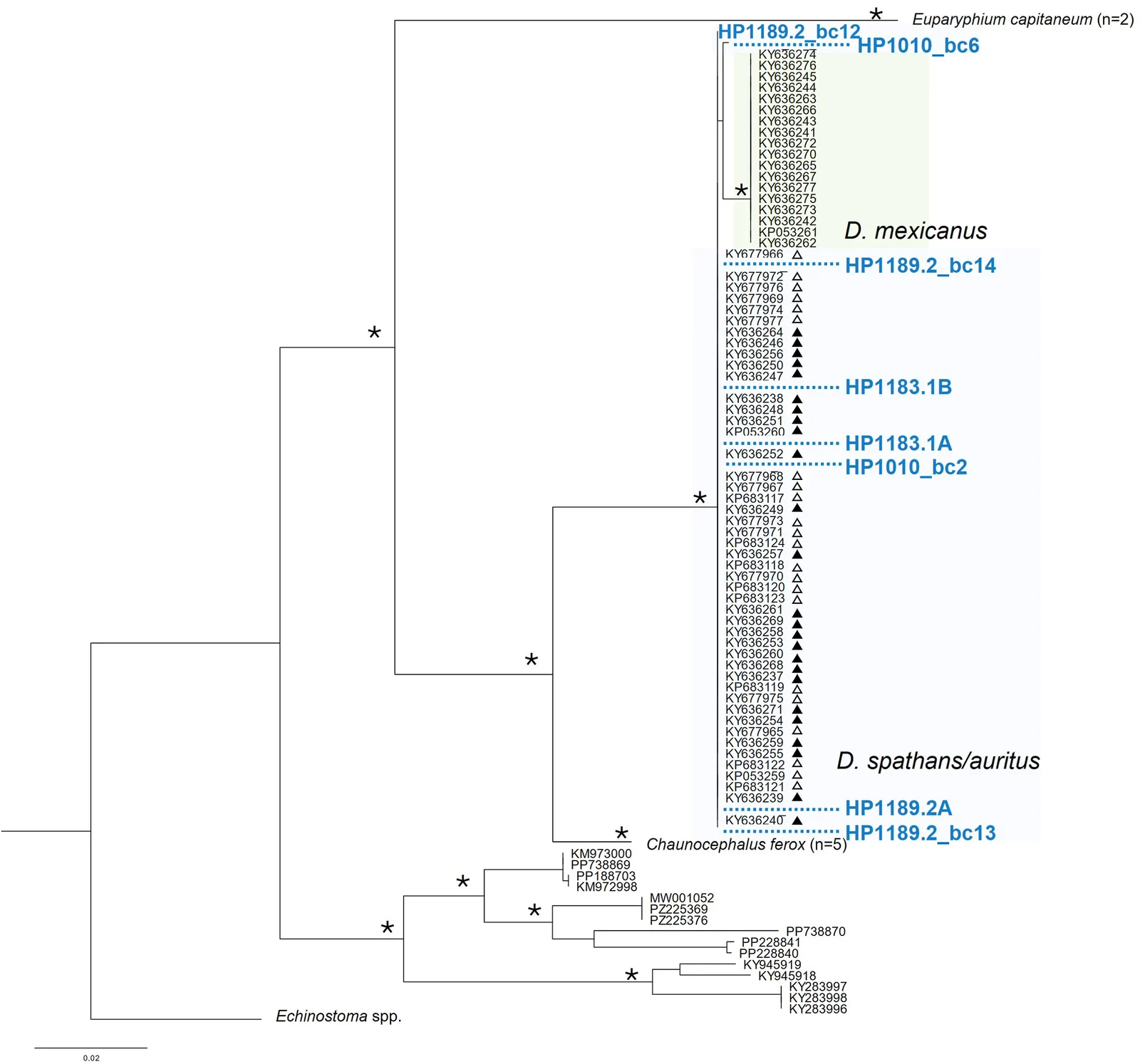
Maximum likelihood (ML) phylogeny inferred from ITS1+5.8S + ITS2 sequences. Bootstrap support values ≥ 70 are indicated by an asterisk (*); exact bootstrap values are reported in the Results. Accession numbers for collapsed clades are provided in Supplementary File 3. Solid triangles indicate sequences designated as *D. spathans,* open triangles indicate sequences designated as *D. auritus*.

Across all loci, phylogenetic analyses recovered short internal branch lengths and shallow genetic divergence throughout the genus. Consistent with Hernández-Cruz et al. (2017), neither *D. spathans* nor *D. auritus* was recovered as a monophyletic taxon. Consequently, all samples falling within the *D. spathans*/*auritus* complex are hereafter referred to as *Drepanocephalus* cf. *spathans*.

Nuclear ribosomal markers (ITS1+5.8S + ITS2 and 28S; Fig. 3, Supplementary File 7) strongly supported the monophyly of *Drepanocephalus* (ITS: bs = 100, pp = 1; 28S: bs = 100, pp = 1). The ITS phylogeny (Fig. 3) strongly supported the monophyly of *D. mexicanus* (bs = 100, pp = 1), whereas the 28S dataset provided only weak support for this relationship (bs = 62, pp = 0.57; Supplementary File 7). Eight sequences generated from *Nannopterum auritus* collected in New York State were included in these analyses. All were confirmed as *Drepanocephalus*; however, one specimen (HP1010_bc6) grouped as sister to *D. mexicanus* without support (ITS: bs = 67, pp = 0.68), whereas all remaining New York specimens grouped within the *D.* cf. *spathans* complex. Despite these placements, nuclear divergence among taxa was extremely low, averaging <0.01% for partial 28S and 0.06% for the ITS region between *D.* cf. *spathans* and *D. mexicanus*. Likewise, HP1010_bc6 differed from other New York specimens by only 0.04% and from *D. mexicanus* by 0.08%. No consistent host-associated or phylogeographic structure was recovered using nuclear markers. The 28S phylogeny (Supplementary File 7) was largely congruent with the ITS tree but, consistent with its more conserved nature, provided lower resolution of species relationships.

The topology of deeper relationships was largely congruent among nuclear and mitochondrial datasets; however, relationships within the *D.* cf. *spathans* complex differed substantially (Fig. 1, Fig. 2, Fig. 3). Both *cox*1 and *nad*1 phylogenies strongly supported two mitochondrial lineages, hereafter referred to as *D.* cf. *spathans* I and *D.* cf. *spathans* II. Placement of *D. mexicanus* differed between analytical methods. Maximum likelihood analyses recovered *D. mexicanus* within the broader *D.* cf. *spathans* complex as sister to either *D.* cf. *spathans* I (*cox*1, Fig. 1) or *D.* cf. *spathans* II (*nad*1, Fig. 2), although support for these relationships was weak or absent (Fig. 1, Fig. 2, Fig. 3). In contrast, Bayesian analyses consistently recovered *D. mexicanus* as sister to the *D.* cf. *spathans* complex with strong support (pp = 1; Supplementary File 8). Bayesian analyses also provided stronger support for the distinction between *D.* cf. *spathans* I and *D.* cf. *spathans* II than did ML analyses.

To further evaluate mitochondrial lineage structure within *Drepanocephalus*, a Generalized Mixed Yule Coalescent (GMYC) analysis was performed using *cox*1 sequences. The single-threshold GMYC model recovered 11 putative clusters, eight of which occurred within the *D.* cf. *spathans* complex (Supplementary File 3). GMYC clusters were largely congruent with mitochondrial phylogenies, with clusters 1 and 2 corresponding to *D.* cf. *spathans* I and clusters 3 and 4 corresponding to *D.* cf. *spathans* II (Table 2). In contrast, these mitochondrial clusters were not consistently recovered by nuclear ribosomal phylogenies (Fig. 3, Supplementary File 7).

**Table 2:** Summary of *Drepanocephalus* mitochondrial lineages, associated definitive hosts, geographic distributions, sample sizes (N), and corresponding *cox*1 haplotype ranges recovered in this study. Clade designations are based on mitochondrial phylogenetic analyses and haplotype network structure. Full accession information for all sequences used can be found in Table 1.

Taxon	Definitive Host	Region	N	Haplotypes
*D.* cf. *spathans* Clade I	*N. auritus*	North America	32	14-29
*D.* cf. *spathans* Clade II	*N. brasilianum*	Mexico/South America	32	30-48
*D. mexicanus*	*N. brasilianum*	Mexico	19	1-13

### 3.4 Genetic diversity and connectivity within *D.* cf. *spathans*

Mitochondrial markers were used to quantify genetic divergence within and among *Drepanocephalus* lineages (Table 3). Within *D.* cf. *spathans* I and II, uncorrected p-distances were <1% for both *cox*1 and *nad*1, although *D.* cf. *spathans* II exhibited slightly greater intraspecific variation. Between *D.* cf. *spathans* I and II, divergence ranged from 3 to 4% for both mitochondrial markers. In contrast, comparisons between either *D.* cf. *spathans* lineage and *D. mexicanus* recovered substantially greater divergence (12–13.9%), with both *D.* cf. *spathans* lineages approximately equidistant from *D. mexicanus*. Nuclear ribosomal markers recovered markedly lower levels of divergence throughout the *D.* cf. *spathans* complex.

**Table 3:** Mean pairwise genetic distances (%) among mitochondrial lineages recovered within the *Drepanocephalus* cf. *spathans* complex based on partial *cox*1 and *nad*1 sequences. Within-lineage comparisons represent average intraspecific divergence, whereas between-lineage comparisons represent average divergence among mitochondrial clades. Abbreviations are as follows: DSI = *Drepanocephalus* cf. *spathans* Clade I; DSII = *Drepanocephalus* cf. *spathans* Clade II; DM = *Drepanocephalus mexicanus*.

Comparison	*cox*1 (%)	*nad*1 (%)
Within *D.* cf. *spathans* I (DSI)	0.405	0.02
Within *D.* cf. *spathans* II (DSII)	0.639	0.825
Within *D. mexicanus* (DM)	1.392	2.647
DSI vs. DSII	3.143	4.086
DSI vs. DM	12.293	13.177
DSII vs. DM	12.706	13.894
Total *D.* cf. *spathans* vs. DM	12.499	13.473

Relationships among unique *Drepanocephalus* haplotypes (Fig. 4) further supported the presence of shallow but persistent mitochondrial structure. A total of 83 *cox*1 sequences were collapsed into 48 haplotypes containing 123 variable sites. Bayesian analyses of haplotypes recovered *D.* cf. *spathans* I and *D.* cf. *spathans* II as strongly supported mitochondrial lineages (pp = 1), with *D. mexicanus* recovered as their sister taxon (pp = 1). Within *D. mexicanus*, two well-supported mitochondrial subclades (pp = 1) corresponded to GMYC clusters 6 and 7. These subclades were consistently recovered in both *cox1* and *nad1* phylogenies but were not recovered in the nuclear ribosomal phylogenies.

**Fig. 4:**
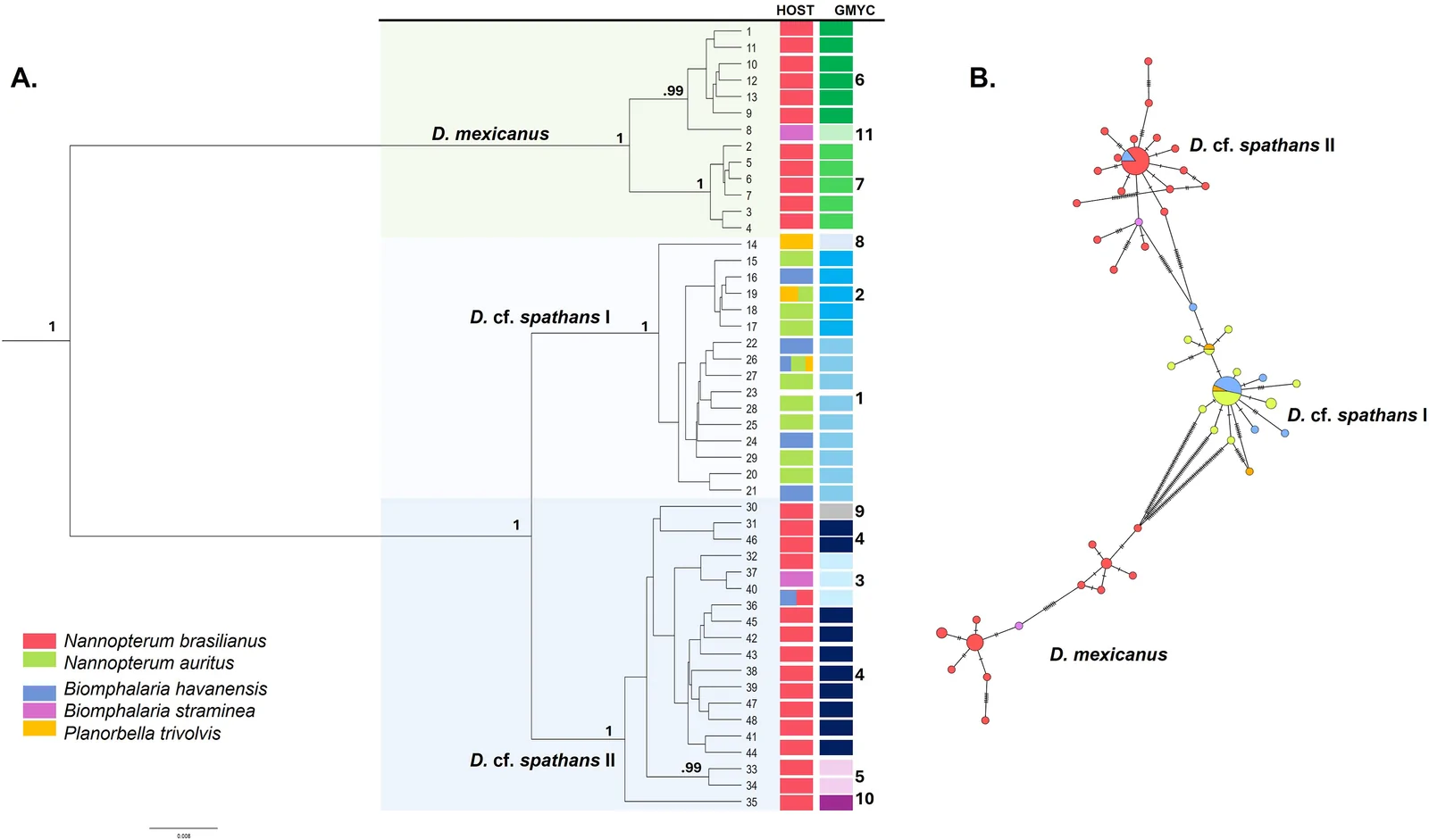
(A) Bayesian phylogeny of all *Drepanocephaluscox*1 sequences collapsed into haplotypes. Posterior probabilities ≥0.98 are indicated at the corresponding nodes. Terminal taxa are labeled by unique haplotype number; corresponding accession numbers are provided in Supplementary File 3. Tips are colored by host taxon (left) and GMYC cluster (right), with the corresponding GMYC cluster number indicated to the right of each tip. (B) Minimum spanning network (MSN) of *Drepanocephaluscox*1 haplotypes, colored by host taxon. Two mitochondrial clades within the *D. spathans*/*auritus* complex were recovered and are designated *D.* cf. *spathans* I and *D.* cf. *spathans* II.

*Drepanocephalus* cf. *spathans* I was recovered from Double-crested Cormorants (*Nannopterum auritus*) and from the planorbid snails *Planorbella trivolvis* and *Biomphalaria havanensis*. In contrast, *D.* cf. *spathans* II was recovered from Neotropic Cormorants (*N. brasilianus*) (Pinto et al., 2016; Hernández-Cruz et al., 2017), and *B. havanensis* (Alberson et al., 2017) and *B. straminea* (Pinto et al., 2016, Fernandez and Laidemitt, 2026).

A minimum spanning network (MSN) of *cox*1 haplotypes was constructed to visualize relationships among mitochondrial lineages (Fig. 4). When haplotypes were colored by host association, lineage membership was strongly partitioned by definitive host, more so than by geographic locality (Fig. 4; Supplementary File 9). Networks colored by host association, geographic locality, and GMYC cluster assignment (respectively, Fig. 4, Supplementary File 9, 10) recovered patterns largely congruent with the mitochondrial phylogenies. *Drepanocephalus*cf. *spathans* I and II formed distinct haplotype groups, while the two *D. mexicanus* mitochondrial subclades corresponded closely with GMYC clusters 6 and 7.

Population genetic summary statistics varied among mitochondrial lineages and GMYC clusters (Table 4). Overall, *D.* cf. *spathans* II exhibited greater nucleotide and haplotype diversity than *D.* cf. *spathans* I, largely because of the low diversity observed within GMYC cluster 1. Comparisons of *nad*1 sequences within *D.* cf. *spathans* II revealed substantial geographic variation; sequences from Argentina and Mexico, representing the presumed southern and northern limits of the lineage, differed by approximately 3.5%. Both *D.* cf. *spathans* I and II exhibited significantly negative Tajima's D values (Table 4).

**Table 4:** Population genetic summary statistics for mitochondrial lineages and GMYC clusters recovered within the *Drepanocephalus* cf. *spathans* complex based on partial *cox*1 sequences. Abbreviations are as follows: n = number of sequences; S = number of segregating sites; π = nucleotide diversity; nhap = number of haplotypes; Hd = haplotype diversity; Theta-W = Watterson's theta; D = Tajima's D. Significant departures from neutrality are indicated as follows: *p < 0.05; **p < 0.01; ***p < 0.001. Singleton GMYC clusters are included for completeness, but population genetic statistics could not be calculated for clusters represented by a single sequence.

Mitochondrial Lineage	n	S	pi	nhap	Hd	Theta-W	D
*D.* cf. *spathans* I	32	17	0.00338	16	0.5	4.455	−2.18131**
GMYC 1	23	7	0.00128	8	0.58	1.454	−2.01499*
GMYC 2	8	12	0.00665	7	0.964	4.082	−1.4567
GMYC 8	1	-	-	-	-	-	-
*D.* cf. *spathans* II	32	102	0.0144	20	0.843	25.327	−2.68528***
GMYC 3	3	5	0.00621	3	1	3.33	-
GMYC 4	25	80	0.01273	13	0.74	21.187	−2.28511**
GMYC 5	2	4	0.00745	2	1	4	-
GMYC 9	1	-	-	-	-	-	-
GMYC 10	1	-	-	-	-	-	-

Across all analyses, mitochondrial markers consistently recovered two major lineages within the *D.* cf. *spathans* complex, whereas nuclear ribosomal markers recovered substantially lower sequence divergence and limited phylogenetic resolution.

## 4 Discussion

### 4.1 Shallow divergence and species boundaries within *Drepanocephalus*

This study expands mitochondrial sampling of the widespread cormorant trematode *Drepanocephalus* cf. *spathans* and provides evidence for the persistence of multiple shallow mitochondrial lineages across the Americas. Collectively, our multilocus analyses support unresolved species boundaries within the *D. spathans/auritus* complex and reveal clear mito-nuclear discordance.

Kudlai et al. (2015) described *Drepanocephalus auritus* based on morphological differences relative to *D. spathans*, including a narrower, more elongate body, an extended uterine field, and more widely separated testes. However, the description was accompanied by limited molecular and phylogenetic analyses. Recent work suggests that these diagnostic characters may not be evolutionarily stable. Alberson et al. (2022) demonstrated substantial morphological plasticity within the *D. spathans/auritus* complex, showing that many of the characters used to distinguish *D. auritus* from *D. spathans* varied with host age, infection intensity, developmental stage, and whether infections were experimental or naturally acquired.

Our multilocus dataset supports broad genetic connectivity across the *D. spathans/auritus* complex while simultaneously revealing previously undescribed host-associated mitochondrial structure (Fig. 4). Although mitochondrial markers consistently recovered two well-supported lineages (Fig. 1, Fig. 2), nuclear ribosomal loci exhibited substantially weaker differentiation and limited phylogenetic resolution (Fig. 3). Collectively, these findings support previous molecular studies (Hernández-Cruz et al., 2017) in demonstrating shallow divergence within the *D.* cf. *spathans* complex. However, the mito-nuclear discordance observed here does not distinguish among ongoing gene flow, incomplete lineage sorting following recent divergence, or incipient speciation. Consequently, the available data are insufficient to determine whether *D.* cf. *spathans* I and II represent population-level mitochondrial structure or independently evolving lineages.

### 4.2 Migratory cormorants and parasite connectivity

These data support a growing body of literature demonstrating that highly vagile hosts can maintain broad parasite connectivity despite shallow lineage structure (Locke et al., 2021; Ebbs et al., 2025; Perrin et al., 2025). Parasite distributions are often shaped by the movements of their most vagile host (Blasco-Costa et al., 2012; Ebbs et al., 2016; Poulin and de Angeli Dutra, 2021). Accordingly, migratory birds provide an effective mechanism for maintaining parasite dispersal and population connectivity across broad geographic scales (Ebbs et al., 2016, 2025; Leung and Koprivnikar, 2016; Perrin et al., 2025). *Drepanocephalus* is a core trematode of Double-crested Cormorants (Sheehan et al., 2017), and its widespread occurrence throughout the host's range is consistent with high levels of continental parasite connectivity.

The Double-crested Cormorant (*N. auritus*) is highly migratory throughout North America, whereas the Neotropic Cormorant (*N. brasilianum*) is largely resident throughout Central and South America, although regional movements do occur. These contrasting movement patterns may help explain the shallow but persistent mitochondrial structure recovered within the *D.* cf. *spathans* complex. Minimum spanning networks and mitochondrial phylogenies suggest that lineage structure corresponded more closely with definitive host association than with geographic locality, although broader geographic and host sampling will be required to test this hypothesis. *Drepanocephalus* cf. *spathans* I has only been recovered from Double-crested Cormorants, whereas *D.* cf. *spathans* II has only been reported from Neotropic Cormorants (Supplementary File 3, Fig. 4). Likewise, *D. mexicanus* has only been reported from the Neotropic Cormorant.

Significant negative Tajima's D values (Table 4) recovered for both mitochondrial clades suggest recent demographic expansion and ongoing parasite connectivity across the Americas. Although these processes reduce opportunities for deep lineage divergence, differences in host ecology and movement patterns may promote partial ecological separation between Nearctic and Neotropical transmission systems. Combined with differences in intermediate-host availability, these ecological factors may facilitate the persistence of shallow mitochondrial lineages despite incomplete reproductive isolation.

Interestingly, parasite surveys of cormorants outside the Americas (Molnár et al., 2015; Cech et al., 2017; Chibwana and Katandukila, 2021) have not reported *Drepanocephalus*. Instead, surveys of *Phalacrocorax carbo* from Eurasia and Africa consistently recover related echinostomatids, primarily species of *Petasiger*, which also utilize *Biomphalaria* and other planorbid snails (Laidemitt et al., 2019; Outa et al., 2020; Hüsken et al., 2026). Kennedy and Spencer (2014) recognized New World (*Nannopterum*) and Old World (*Phalacrocorax*) cormorants as distinct genera based on molecular phylogenetic evidence. Together, these observations suggest that the evolutionary history of *Drepanocephalus* may be closely tied to New World cormorant–planorbid transmission systems rather than to cormorants globally. In contrast, *Hysteromorpha* spp. (Diplostomidae), which also cycle between planorbid snails and cormorants, exhibit mitochondrial evidence of continentally restricted species, including a distinct Old World representative (Achatz et al., 2025).

### 4.3 Intermediate host associations and ecological structuring of mitochondrial lineages

Colonial breeding by cormorants can facilitate parasite transmission and mixing when suitable intermediate hosts are present. Although records of *Drepanocephalus* from first intermediate hosts remain limited, available studies demonstrate that transmission occurs through multiple planorbid snail species, including *Biomphalaria havanensis*, *B. straminea*, and *Planorbella trivolvis* (Griffin et al., 2012; Pinto et al., 2016; Gordy and Hanington, 2019; Alberson et al., 2017; Hernández-Cruz et al., 2017). These snail species differ substantially in their geographic distributions and ecological associations, potentially creating regional differences in parasite transmission opportunities.

Among molecularly characterized records currently available, *D.* cf. *spathans* I has been recovered from *B. havanensis* and *P. trivolvis*, whereas *D.* cf. *spathans* II has been recovered from *B. havanensis* and *B. straminea* (Fig. 4). Although these records remain sparse, the currently documented host associations differ between mitochondrial lineages and are consistent with the hypothesis that differences in intermediate-host availability may contribute to the maintenance of shallow mitochondrial structure across the geographic range of *D.* cf. *spathans*.

Interestingly, *D. mexicanus* has also been reported from *B. straminea*, indicating that portions of the geographic ranges of *D. mexicanus* and *D.* cf. *spathans* II overlap in both intermediate and definitive host use. Despite this ecological overlap, *D. mexicanus* remained distinct from the *D.* cf. *spathans* complex in all mitochondrial analyses. However, the limited divergence recovered from nuclear markers suggests that species boundaries within *Drepanocephalus* remain incompletely resolved.

### 4.4 Implications for parasite transmission and aquaculture

*Biomphalaria havanensis* and *Planorbella trivolvis* both occur in catfish aquaculture facilities in the southeastern United States (George, 2008; Griffin et al., 2012), while Double-crested Cormorants increasingly exploit these systems as foraging habitats (King et al., 2010; Scherr et al., 2010; Burr et al., 2020). Together, these conditions create ecological interfaces that facilitate *Drepanocephalus* transmission between definitive, intermediate, and second intermediate hosts (Sheehan et al., 2017). Griffin et al. (2012) demonstrated that *Drepanocephalus* infections can cause substantial pathology and mortality in juvenile channel catfish, highlighting the economic importance of understanding parasite transmission within aquaculture systems.

Our phylogenetic analyses suggest that outbreaks reported from catfish aquaculture facilities in the southeastern United States were most likely associated with *D.* cf. *spathans* I, the lineage currently associated with the highly migratory Double-crested Cormorant and the broadly distributed planorbid snails *P. trivolvis* and *B. havanensis*. Although this hypothesis requires confirmation through additional sequencing of historical and contemporary aquaculture isolates, it illustrates how improved parasite systematics can inform disease surveillance and transmission studies.

To our knowledge, *Drepanocephalus* spp. have not yet been reported from snail intermediate hosts in New York State. Moreover, apart from surveys conducted in Alberta, Canada (Gordy and Hanington, 2019; McPhail et al., 2025), records of naturally infected intermediate hosts remain scarce throughout North America. Expanding surveys of planorbid snails, together with molecular characterization of larval *Drepanocephalus*, will improve our understanding of transmission ecology and help identify regions where aquaculture systems may be vulnerable to parasite emergence.

## 5 Conclusion

This study used multilocus phylogenetic and genomic data to evaluate the placement of *Drepanocephalus* collected from New York State and to investigate phylogeographic patterns within the geographically widespread *D.* cf. *spathans* complex. Our results indicate that the *D.* cf. *spathans* complex occurs across the Americas and comprises shallow but structured mitochondrial lineages that are likely associated with host use. The shallow mitochondrial divergence and limited nuclear differentiation observed here are consistent with recent divergence accompanied by incomplete lineage sorting, ongoing gene flow among transmission systems, or a combination of both processes. The observed mitochondrial structure is further consistent with partially distinct Nearctic and Neotropical transmission systems shaped by host movement ecology and intermediate-host distributions. Future studies should expand sampling throughout South America, particularly within the southern range of *D.* cf. *spathans* II and generate complete mitochondrial genomes from additional lineages to better resolve mitochondrial divergence, lineage demographics, gene flow, and species boundaries. Together, these findings demonstrate how host movement ecology and transmission networks can maintain shallow but persistent parasite population structure across continental scales despite limited nuclear differentiation.

## CRediT authorship contribution statement

**Daniel Muñoz:** Writing – review & editing, Writing – original draft, Visualization, Methodology, Formal analysis, Data curation. **Stephen E. Harris:** Writing – review & editing, Resources, Methodology, Funding acquisition. **Erika T. Ebbs:** Writing – review & editing, Writing – original draft, Visualization, Validation, Supervision, Resources, Project administration, Methodology, Investigation, Funding acquisition, Formal analysis, Data curation, Conceptualization.

## Data availability

All nucleotide sequence data generated in this study have been deposited in GenBank under the BioProject PRJNA1518865. Voucher host and parasite specimens have been accessioned into the Museum of Southwestern Biology, Division of Parasites (MSB:Para:54101–54129) and are publicly accessible through the Arctos database (https://arctos.database.museum). Associated specimen metadata, including host identity, collection locality, and voucher information, are available through the corresponding GenBank and Arctos records.

## Funding

This work was primarily supported by the National Science Foundation (NSF), USA, through award DBI-2218190 to E.T.E. and S.E.H.

## Declaration of competing interest

The authors declare that they have no known competing financial interests or personal relationships that could have appeared to influence the work reported in this paper.
