# Supplementary material for: Host-associated mitochondrial structure and broad geographic connectivity in Drepanocephalus associated with migratory cormorants
Source: Int J Parasitol Parasites Wildl. 2026 Sep 18;31:101290. doi: 10.1016/j.ijppaw.2026.101290 (PMC13628594; doi:10.1016/j.ijppaw.2026.101290)
Supplement: Supplementary file 1 [file mmc1.docx]

**Supplementary File 1.** Summary of helminths recovered from 15 Double-crested Cormorants (*Nannopterum auritus*) necropsied in this study. n.c. = not counted. A subset of *Drepanocephalus* specimens was used for Oxford Nanopore Technologies (ONT) whole-genome sequencing in undergraduate Genetics CUREs conducted in Spring 2024 (HP1010; *n* = 8) and Spring 2026 (HP1189; *n* = 4).

| **Specimen ID** | **Host Species** | ***Drepanocephalus*** | **Intensity** | **Other Helminths Recovered** |
| --- | --- | --- | --- | --- |
| HP1004 | *N. auritus* | MSB:Para:54101 | n.c. | Cyclophyllidea sp. (MSB:Para:54102) |
| HP1008 | *N. auritus* | MSB:Para:54103 | 80 | - |
| HP1010 | *N. auritus* | MSB:Para:54105 | n.c. | Schistosomatidae sp. (MSB:Para:54104); Cyclophyllidea sp. (MSB:Para:54107);     Nematoda sp. (MSB:Para:54106) |
| HP1011 | *N. auritus* | - | 0 | - |
| HP1182 | *N. auritus* | MSB:Para:54108 | 11 | - |
| HP1183 | *N. auritus* | MSB:Para:54109 | 3 | Cyclophyllidea sp. (MSB:Para:54110) |
| HP1184 | *N. auritus* | - | 0 | - |
| HP1185 | *N. auritus* | - | 0 | Trematoda sp. (MSB:Para:54111);           Nematoda sp. (MSB:Para:54112) |
| HP1186 | *N. auritus* | - | 0 | Schistosomatidae sp. (MSB:Para:54113); Trematoda sp. (MSB:Para:54115);     Cyclophyllidea sp. (MSB:Para:54114) |
| HP1187 | *N. auritus* | - | 0 | Echinostomatidae sp. (MSB:Para:54117); Cyclophyllidea sp. (MSB:Para:54116) |
| HP1188 | *N. auritus* | - | 0 | Trematoda sp. (MSB:Para:54119);     Cyclophyllidea sp. (MSB:Para:54118) |
| HP1189 | *N. auritus* | MSB:Para:54121 | 3 | Cyclophyllidea sp. (MSB:Para:54120) |
| HP1190 | *N. auritus* | MSB:Para:54123 | 0 | Hysteromorpha sp. (MSB:Para:54123); Cyclophyllidea sp. (MSB:Para:54122) |
| HP1191 | *N. auritus* | - | 0 | Cyclophyllidea sp. (MSB:Para:54125) |
| HP1192 | *N. auritus* | MSB:Para:54126 | 25 | Hysteromorpha sp. (MSB:Para:54128); Cyclophyllidea sp. (MSB:Para:54129);     Nematoda sp. (MSB:Para:54127) |

**Supplementary File 2:** Published occurrences of *Drepanocephalus* spp. and associated host records from the Americas. Shown are species identifications, life stage (L.S), host species, and collection localities reported in the literature and in the present study. ‘n’ refers to the reported collection effort of hosts sampled. Additional citations not included in-text; (a) Flowers, J.R., Poore, M.F., Mullen, J.E., Levy, M.G., 2004. Digeneans Collected from Piscivorous Birds in North Carolina, U.S.A. Comparative Parasitology 71, 243–244. (b) Kostadinova, A., Vaucher, C., Gibson, D.I., 2002. Redescriptions of two echinostomes from birds in Paraguay, with comments on *Drepanocephalus* Dietz, 1909 and Paryphostomum Dietz, 1909 (Digenea: Echinostomatidae). Syst Parasitol 53, 147–158. (c) Wagner, B.A., Hoberg, E.P., Somers, C.M., Soos, C., Fenton, H., Jenkins, E.J., 2012. Gastrointestinal Helminth Parasites of Double-Crested Cormorants (*Phalacrocorax* *auritus*) at Four Sites in Saskatchewan, Canada. Comparative Parasitology 79, 275–282.

| **Taxa** | **Collectors** | **L.S** | **Host Species** | **Localities** | **n** |
| --- | --- | --- | --- | --- | --- |
| *Drepanocephalus spathans* | Dietz, 1909 | A | *Nannopterum brasilianus, Nannopterum auritus* | Brazil | n.r. |
| *Drepanocephalus spathans* | Threlfall, 1982 | A | *Nannopterum auritus* | USA: Florida | 42 |
| *Drepanocephalus spathans* | Fedynich et al. , 1997 | A | *Nannopterum brasilianus, Nannopterum auritus* | USA: Texas | 12 |
| *Drepanocephalus spathans* | Kostadinova et al., 2002 | A | *Nannopterum brasilianus* | Paraguay | 1 |
| *Drepanocephalus spathans* | Flowers et al., 2004 | A | *Nannopterum auritus* | USA: North Carolina | 7 |
| *Drepanocephalus spathans* | Drago et al., 2011 | A | *Nannopterum brasilianus* | Argentina | 13 |
| *Drepanocephalus spathans* | Monteiro et al., 2011 | A | *Nannopterum brasilianus* | Brazil | 47 |
| *Drepanocephalus spathans* | Griffin et. al, 2012 | C | *Planorbella trivolvis* | USA: Mississippi | n.r. |
| *Drepanocephalus spathans* | Wagner et al., 2012 | A | *Nannopterum auritus* | Canada: Saskatchewan | 92 |
| *Drepanocephalus spathans* | O'Hear et al., 2014 | A | *Nannopterum auritus* | USA: Mississippi | 35 |
| *Drepanocephalus spathans* | Sheehan et al., 2013 | A | *Nannopterum auritus* | USA: Alabama, Minnesota, Mississippi, Vermont | 218 |
| *Drepanocephalus spathans* | Gordy et al., 2019 | C | *Planorbella trivolvis* | Canada: Alberta | n.r. |
| *Drepanocephalus spathans* | Van Steenkiste et al., 2015 | A | *Nannopterum auritus* | Canada: Ontario | n.r. |
| *Drepanocephalus spathans* | Hernandez-Cruz et al., 2017 | A | *Nannopterum brasilianus, Nannopterum auritus* | Mexico | 61 |
| *Drepanocephalus spathans* | Gordy & Hanington, 2019 | C | *Planorbella trivolvis* | Canada: Alberta | 1166 |
| *Drepanocephalus spathans* | Rietschel, G., & Werding, B., 1978 | A | *Nannopterum brasilianus, Sula leucogaster* | Colombia | 3 |
| *Drepanocephlaus cf. spathans* | This Study | A | *Nannopterum auritus* | USA: New York | 15 |
| *Drepanocephalus auritus* | Kudlai et al., 2015 | A | *Nannopterum auritus* | USA: North Dakota, Mississippi, Minnesota | n.r. |
| *Drepanocephalus auritus* | Alberson et al., 2017 | C | *Biomphalaria havanensis* | USA: Mississippi | 1740 |
| *Drepanocephalus mexicanus* | Lamothe-Argumendo & Pérez Ponce de Léon, 1989 | A | *Nannopterum brasilianus* | Mexico | n.r. |
| *Drepanocephalus mexicanus* | Hernandez-Cruz et al., 2017 | A | *Nannopterum brasilianus* | Mexico | 61 |

**Supplementary File 4:**  Mitochondrial genome assemblies for three individual *Drepanocephalus* specimens. Visualized in Geneious Prime.

**Supplementary File 5:** MITOS annotation of *Drepanocephalus* cf. *spathans* mitochondrial genome, including 12 protein coding genes, 22 tRNAs and 2 rRNAs. ‘# of polymorphisms' refers to the number of intraspecific nucleotide differences between the three assembled mitochondrial genomes (HP1183.1A, 1B and HP1189.2)

|  | **Type** | **Minimum** | **Maximum** | **Length** | **# polymorphic sites** |
| --- | --- | --- | --- | --- | --- |
| **tRNA-Glu** | tRNA | 13641 | 13703 | 63 |  |
| **tRNA-Gly** | tRNA | 13556 | 13619 | 64 |  |
| **ND5** | CDS | 11964 | 13544 | 1581 | 11 |
| **tRNA-Arg** | tRNA | 11901 | 11961 | 61 |  |
| **tRNA-Leu** | tRNA | 11831 | 11893 | 63 |  |
| **tRNA-Ser** | tRNA | 11761 | 11826 | 66 |  |
| **tRNA-Leu** | tRNA | 11701 | 11764 | 64 |  |
| **tRNA-Tyr** | tRNA | 11637 | 11700 | 64 |  |
| **ND6** | CDS | 11181 | 11636 | 456 | 1 |
| **COX2** | CDS | 10502 | 11179 | 678 | 6 |
| **12S rRNA** | rRNA | 9748 | 10478 | 731 |  |
| **tRNA-Cys** | tRNA | 9683 | 9747 | 65 |  |
| **16S rRNA** | rRNA | 8708 | 9682 | 975 |  |
| **tRNA-Thr** | tRNA | 8642 | 8708 | 67 |  |
| **COX1** | CDS | 7062 | 8615 | 1554 | 10 |
| **tRNA-Trp** | tRNA | 6996 | 7058 | 63 |  |
| **tRNA-Ser** | tRNA | 6923 | 6980 | 58 |  |
| **ND3** | CDS | 6559 | 6915 | 357 |  |
| **tRNA-Lys** | tRNA | 6493 | 6556 | 64 | 2 |
| **tRNA-Ile** | tRNA | 6419 | 6483 | 65 |  |
| **tRNA-Pro** | tRNA | 6347 | 6416 | 70 |  |
| **tRNA-Asn** | tRNA | 6273 | 6343 | 71 |  |
| **ND1** | CDS | 5371 | 6273 | 903 | 2 |
| **tRNA-Asp** | tRNA | 5301 | 5366 | 66 |  |
| **tRNA-Ala** | tRNA | 5230 | 5297 | 68 |  |
| **tRNA-Val** | tRNA | 5142 | 5205 | 64 |  |
| **ND2** | CDS | 4272 | 5141 | 870 | 9 |
| **ATP6** | CDS | 3750 | 4265 | 516 | 5 |
| **tRNA-Met** | tRNA | 3686 | 3749 | 64 |  |
| **tRNA-Phe** | tRNA | 3612 | 3676 | 65 |  |
| **tRNA-Gln** | tRNA | 3547 | 3609 | 63 |  |
| **ND4** | CDS | 2259 | 3539 | 1281 | 6 |
| **ND4L** | CDS | 2035 | 2298 | 264 | 1 |
| **CYTB** | CDS | 912 | 2027 | 1116 | 7 |
| **tRNA-His** | tRNA | 848 | 911 | 64 |  |
| **COX3** | CDS | 199 | 843 | 645 | 3 |

**Supplementary File 6:** Mitochondrial genome summary of Echinostomatids compared with recovered *Drepanocephalus* cf. *spathans* mitochondrial chromosome. All sampled trematode mitochondrial genomes contained 12 protein-coding genes, consistent with the apparent absence of atp8 in most digeneans.

| **Taxa** | **Accession** | **Genome Size** | **GC%** | **PCGs** | **tRNAs** | **Circularized** | **Reference** |
| --- | --- | --- | --- | --- | --- | --- | --- |
| *Artyfechinostomum malayanum* | OK509083 | 17175 | 37.7 | 12 | 22 | yes | Li et al. 2018 |
| *Echinostoma revolutum* | MN116706 | 15714 | 38.3 | 12 | 22 | yes | Ran et al. 2020 |
| *Echinoparyphium aconiatum* | ON644993 | 14865 | 35.7 | 12 | 22 | yes | Gacad et al. 2023 |
| *Hypoderaeum conoideum* | PP110501 | 18011 | 38.5 | 12 | 22 | yes | Pham et al. 2024 |
| *Chaunocephalus ferox* | PV685907 | 17482 | 38.6 | 12 | 22 | yes | Zhou et al. 2025 |
| *Drepanocephalus* cf. *spathans* | PRJNA1518865 | 13,818 | 37.4 | 12 | 22 | no | This study |

**Supplemental File 7:** Maximum likelihood phylogeny of partial 28S rRNA (1,025bp) sequences from *Drepanocephalus* spp. and representative echinostomatid outgroups. Newly generated sequences from New York specimens are highlighted in blue. Shaded regions indicate the *Drepanocephalus* cf. *spathans* complex and *D. mexicanus* clade. Relevant bootstrap support values are indicated on the appropriate branches. Scale bar indicates substitutions per site.

**Supplemental File 8:** Maximum Likelihood analysis of partial *cox*1 sequences from *Drepanocephalus* spp. and representative echinostomatid outgroups. Newly generated sequences from New York specimens are highlighted in blue. Shaded regions indicate the *Drepanocephalus* cf. *spathans* complex and *D. mexicanus* clade. Relevant bootstrap support values are indicated on the appropriate branches. Scale bar indicates substitutions per site.

**Supplementary File 9:**Minimum Spanning Network of *Drepanocephalus* spp. labeled by geography.

**Supplementary File 10:** Minimum Spanning Network of *Drepanocephalus* spp. labeled by GMYC cluster.
