# Supplementary material for: Host-associated mitochondrial structure and broad geographic connectivity in Drepanocephalus associated with migratory cormorants
Source: Int J Parasitol Parasites Wildl. 2026 Sep 18;31:101290. doi: 10.1016/j.ijppaw.2026.101290 (PMC13628594; doi:10.1016/j.ijppaw.2026.101290)
Supplement: Supplementary file 2 [file mmc2.docx]

| Taxa & Collector ID | Host | Locality | CO1 Haplotype | GMYC Cluster | CO1 | ND1 | 28S | ITS1-5.8S-ITS2 | Source |
| --- | --- | --- | --- | --- | --- | --- | --- | --- | --- |
| *Drepanocephalus auritus CD7* | *Biomphalaria havanensis* | USA: Mississippi | Hap_26 | 1 | KY677958 | KY677945 | KY677971 | KY677971 | Alberson et al. 2017 |
| *Drepanocephalus auritus CD8* | *Biomphalaria havanensis* | USA: Mississippi | Hap_26 | 1 | KY677959 | KY677946 | KY677972 | - | Alberson et al. 2017 |
| *Drepanocephalus auritus WD1* | *Biomphalaria havanensis* | USA: Mississippi | Hap_26 | 1 | KY677960 | KY677947 | KY677973 | KY677973 | Alberson et al. 2017 |
| *Drepanocephalus auritus WD3* | *Biomphalaria havanensis* | USA: Mississippi | Hap_26 | 1 | KY677961 | KY677948 | KY677974 | KY677974 | Alberson et al. 2017 |
| *Drepanocephalus auritus WD4* | *Biomphalaria havanensis* | USA: Mississippi | Hap_36 | 4 | KY677962 | KY677949 | KY677975 | KY677975 | Alberson et al. 2017 |
| *Drepanocephalus auritus WD5* | *Biomphalaria havanensis* | USA: Mississippi | Hap_26 | 1 | KY677963 | KY677950 | KY677976 | KY677976 | Alberson et al. 2017 |
| *Drepanocephalus auritus CD6* | *Biomphalaria havanensis* | USA: Mississippi | Hap_26 | 1 | KY677957 | KY677944 | KY677970 | KY677970 | Alberson et al. 2017 |
| *Drepanocephalus auritus CD5* | *Biomphalaria havanensis* | USA: Mississippi | Hap_26 | 1 | KY677956 | KY677943 | KY677969 | KY677969 | Alberson et al. 2017 |
| *Drepanocephalus auritus WD6* | *Biomphalaria havanensis* | USA: Mississippi | Hap_24 | 1 | KY677964 | KY677951 | KY677977 | KY677977 | Alberson et al. 2017 |
| *Drepanocephalus auritus CD4* | *Biomphalaria havanensis* | USA: Mississippi | Hap_16 | 2 | KY677955 | KY677942 | KY677968 | KY677968 | Alberson et al. 2017 |
| *Drepanocephalus auritus CD3* | *Biomphalaria havanensis* | USA: Mississippi | Hap_21 | 2 | KY677954 | KY677941 | KY677967 | KY677967 | Alberson et al. 2017 |
| *Drepanocephalus auritus CD1* | *Biomphalaria havanensis* | USA: Mississippi | Hap_36 | 4 | KY677952 | KY677939 | KY677965 | KY677965 | Alberson et al. 2017 |
| *Drepanocephalus auritus CD2* | *Biomphalaria havanensis* | USA: Mississippi | Hap_22 | 1 | KY677953 | KY677940 | KY677966 | KY677966 | Alberson et al. 2017 |
| *Drepanocephalus auritus MGC1704* | *Planorbella trivolvis* | Canada: Alberta | Hap_19 | 2 | KT831381 | - | - | - | Gordy et al. 2015 |
| *Drepanocephalus auritus 1* | *Nannopterum auritus* | USA: North Dakota | Hap_26 | 1 | KP683125 | - | KP683117 | KP683117 | Kudlai et al. 2015 |
| *Drepanocephalus auritus 2* | *Nannopterum auritus* | USA: North Dakota | Hap_26 | 1 | KP683126 | - | KP683118 | KP683118 | Kudlai et al. 2015 |
| *Drepanocephalus auritus 3* | *Nannopterum auritus* | USA: North Dakota | Hap_26 | 1 | KP683127 | - | KP683119 | KP683119 | Kudlai et al. 2015 |
| *Drepanocephalus auritus 4* | *Nannopterum auritus* | USA: Minnesota | Hap_28 | 1 | KP683128 | - | KP683120 | KP683120 | Kudlai et al. 2015 |
| *Drepanocephalus auritus 5* | *Nannopterum auritus* | USA: Minnesota | Hap_20 | 2 | KP683129 | - | KP683121 | KP683121 | Kudlai et al. 2015 |
| *Drepanocephalus auritus 6* | *Nannopterum auritus* | USA: Minnesota | Hap_26 | 1 | KP683130 | - | KP683122 | KP683122 | Kudlai et al. 2015 |
| *Drepanocephalus auritus 7* | *Nannopterum auritus* | USA: Mississippi | Hap_27 | 1 | KP683131 | - | KP683123 | KP683123 | Kudlai et al. 2015 |
| *Drepanocephalus auritus 8* | *Nannopterum auritus* | USA: Mississippi | Hap_27 | 1 | KP683132 | - | KP683124 | KP683124 | Kudlai et al. 2015 |
| *Drepanocephalus auritus EC.IN.Pa.22.V27.19* | *Nannopterum auritus* | Canada: Ontario | Hap_23 | 1 | KM538090 | - | - | - | Van Steenkiste et al. 2015 |
| *Drepanocephalus auritus MJG-DA* | *Planorbella trivolvis* | USA | Hap_26 | 1 | KR259644 | KP053262 | KP053259 | KP053259 | Pinto et al. 2016 |
| *Drepanocephalus auritus HAP-H1* | *Biomphalaria straminea* | Brazil | Hap_37 | 3 | KP053255 | KP053263 | KP053260 | KP053260 | Pinto et al. 2016 |
| *Drepanocephalus auritus MGC1762* | *Planorbella trivolvis* | Canada: Alberta | Hap_14 | 8 | MH369294 | - | - | - | Gordy and Hannington 2019 |
|  |  |  |  |  |  |  |  |  |  |
| *Drepanocephalus spathans DNA2624* | *Nannopterum brasilianus* | Mexico | Hap_35 | 10 | KY636190 | KY636288 | - | KY636271 | Hernandez-Cruz et al 2017 |
| Drepanocephalus spathans DNA2621 | *Nannopterum brasilianus* | Mexico | Hap_32 | 3 | KY636192 | - | - | KY636269 | Hernandez-Cruz et al 2017 |
| *Drepanocephalus spathans DNA1528* | *Nannopterum brasilianus* | Mexico | Hap_31 | 4 | KY636233 | - | - | - | Hernandez-Cruz et al 2017 |
| *Drepanocephalus spathans DNA2619* | *Nannopterum brasilianus* | Mexico | Hap_30 | 9 | KY636194 | KY636287 | - | KY636268 | Hernandez-Cruz et al 2017 |
| *Drepanocephalus spathans DNA1511* | *Nannopterum auritus* | Mexico | Hap_15 | 2 | KY636232 | - | - | - | Hernandez-Cruz et al 2017 |
| *Drepanocephalus spathans DNA1510* | *Nannopterum auritus* | Mexico | Hap_17 | 2 | KY636231 | - | - | - | Hernandez-Cruz et al 2017 |
| *Drepanocephalus spathans DNA1512* | *Nannopterum auritus* | Mexico | Hap_19 | 2 | KY636219 | - | - | - | Hernandez-Cruz et al 2017 |
| *Drepanocephalus spathans DNA1513* | *Nannopterum auritus* | Mexico | Hap_26 | 1 | KY636220 | KY636289 | - | KY636237 | Hernandez-Cruz et al 2017 |
| *Drepanocephalus spathans DNA1514* | *Nannopterum auritus* | Mexico | Hap_29 | 1 | KY636221 | - | - | KY636238 | Hernandez-Cruz et al 2017 |
| *Drepanocephalus spathans DNA1515* | *Nannopterum auritus* | Mexico | Hap_26 | 1 | KY636222 | - | - | KY636239 | Hernandez-Cruz et al 2017 |
| *Drepanocephalus spathans DNA2321* | *Nannopterum brasilianus* | Mexico | - | - | - | KY636286 | - | KY636264 | Hernandez-Cruz et al 2017 |
| *Drepanocephalus spathans DNA2322* | *Nannopterum brasilianus* | Mexico | Hap_33 | 5 | KY636200 | - | - | - | Hernandez-Cruz et al 2017 |
| *Drepanocephalus spathans DNA2048* | *Nannopterum brasilianus* | Mexico | Hap_34 | 5 | KY636203 | KY636285 | MF351546 | KY636261 | Hernandez-Cruz et al 2017 |
| *Drepanocephalus spathans DNA2625* | *Nannopterum brasilianus* | Mexico | Hap_36 | 4 | KY636189 | - | - | - | Hernandez-Cruz et al 2017 |
| *Drepanocephalus spathans DNA1540* | *Nannopterum brasilianus* | Mexico | Hap_38 | 4 | KY636207 | KY636294 | - | KY636257 | Hernandez-Cruz et al 2017 |
| *Drepanocephalus spathans DNA1518* | *Nannopterum auritus* | Mexico | Hap_39 | 4 | KY636224 | - | - | - | Hernandez-Cruz et al 2017 |
| *Drepanocephalus spathans DNA1531* | *Nannopterum brasilianus* | Mexico | Hap_40 | 3 | KY636216 | KY636291 | - | KY636250 | Hernandez-Cruz et al 2017 |
| *Drepanocephalus spathans DNA1525* | *Nannopterum brasilianus* | Mexico | Hap_41 | 4 | KY636218 | - | - | KY636247 | Hernandez-Cruz et al 2017 |
| *Drepanocephalus spathans DNA1534* | *Nannopterum brasilianus* | Mexico | Hap_42 | 4 | KY636213 | KY636292 | - | KY636253 | Hernandez-Cruz et al 2017 |
| *Drepanocephalus spathans DNA1535* | *Nannopterum brasilianus* | Mexico | Hap_43 | 4 | KY636212 | - | - | KY636254 | Hernandez-Cruz et al 2017 |
| *Drepanocephalus spathans DNA1537* | *Nannopterum brasilianus* | Mexico | Hap_44 | 4 | KY636210 | - | - | - | Hernandez-Cruz et al 2017 |
| *Drepanocephalus spathans DNA1541* | *Nannopterum brasilianus* | Mexico | Hap_45 | 4 | KY636206 | - | - | KY636258 | Hernandez-Cruz et al 2017 |
| *Drepanocephalus spathans DNA1544* | *Nannopterum brasilianus* | Mexico | Hap_46 | 4 | KY636205 | - | - | - | Hernandez-Cruz et al 2017 |
| *Drepanocephalus spathans DNA2176* | *Nannopterum brasilianus* | Mexico | Hap_47 | 4 | KY636201 | - | - | - | Hernandez-Cruz et al 2017 |
| *Drepanocephalus spathans DNA2620* | *Nannopterum brasilianus* | Mexico | Hap_48 | 4 | KY636193 | - | - | - | Hernandez-Cruz et al 2017 |
| *Drepanocephalus spathans DNA2622* | *Nannopterum brasilianus* | Mexico | Hap_36 | 4 | KY636191 | - | - | - | Hernandez-Cruz et al 2017 |
| *Drepanocephalus spathans DNA1545* | *Nannopterum brasilianus* | Mexico | Hap_36 | 4 | KY636204 | - | - | - | Hernandez-Cruz et al 2017 |
| *Drepanocephalus spathans DNA1539* | *Nannopterum brasilianus* | Mexico | Hap_36 | 4 | KY636208 | - | - | - | Hernandez-Cruz et al 2017 |
| *Drepanocephalus spathans DNA1538* | *Nannopterum brasilianus* | Mexico | Hap_36 | 4 | KY636209 | KY636293 | - | KY636256 | Hernandez-Cruz et al 2017 |
| *Drepanocephalus spathans DNA1536* | *Nannopterum brasilianus* | Mexico | Hap_36 | 4 | KY636211 | - | - | KY636255 | Hernandez-Cruz et al 2017 |
| *Drepanocephalus spathans DNA1533* | *Nannopterum brasilianus* | Mexico | Hap_36 | 4 | KY636214 | - | - | KY636252 | Hernandez-Cruz et al 2017 |
| *Drepanocephalus spathans DNA1532* | *Nannopterum brasilianus* | Mexico | Hap_36 | 4 | KY636215 | - | - | KY636251 | Hernandez-Cruz et al 2017 |
| *Drepanocephalus spathans DNA1526* | *Nannopterum brasilianus* | Mexico | Hap_36 | 4 | KY636217 | - | - | KY636248 | Hernandez-Cruz et al 2017 |
| *Drepanocephalus spathans DNA1516* | *Nannopterum auritus* | Mexico | Hap_36 | 4 | KY636223 | KY636290 | - | KY636240 | Hernandez-Cruz et al 2017 |
| *Drepanocephalus spathans DNA1524* | *Nannopterum brasilianus* | Mexico | Hap_36 | 4 | KY636230 | KY636284 | - | KY636246 | Hernandez-Cruz et al 2017 |
| *Drepanocephalus spathans DNA2047* | *Nannopterum brasilianus* | Mexico | Hap_36 | 4 | KY636234 | - | - | KY636260 | Hernandez-Cruz et al 2017 |
| Drepanocephalus spathans DNA1112 | *Nannopterum brasilianus* | Mexico | - | - | - | - | MF351545 | - | Hernandez-Cruz et al 2017 |
| Drepanocephalus spathans DNA1527 | *Nannopterum brasilianus* | Mexico | - | - | - | - | - | KY636249 | Hernandez-Cruz et al 2017 |
| Drepanocephalus spathans DNA1542 | *Nannopterum brasilianus* | Mexico | - | - | - | - | - | KY636259 | Hernandez-Cruz et al 2017 |
| *Drepanocephalus spathans MGC2353* | *Planorbella trivolvis* | Canada: Alberta | - | - | - | MH368952 | - | - | Gordy and Hannington, 2019 |
| *Drepanocephalus spathans MGC2147* | *Planorbella trivolvis* | Canada: Alberta | - | - | - | MH368951 | - | - | Gordy and Hannington, 2019 |
| *Drepanocephalus spathans BMC208* | *Planorbella trivolvis* | Canada: Alberta | - | - | - | PV595372 | - | - | McPhail et al. 2025 |
| *Drepanocephalus spathans BMC1127* | *Planorbella trivolvis* | Canada: Alberta | - | - | - | PV595384 | - | - | McPhail et al. 2025 |
| *Drepanocephalus spathans MSB:Para:27859* | *Sula leucogaster* | USA: New Mexico | - | - | - | OQ249636 | - | - | Baumann et al. 2024 |
| Drepanocephalus spathans YCA | *Phalacrocorax auritus* | USA: Mississippi | - | - | - | - | JN993269 | - | Griffin et al. 2012 |
| Drepanocephalus spathans HCC | *Planorbella trivolvis* | USA: Mississippi | - | - | - | - | JN993270 | - | Griffin et al. 2012 |
|  |  |  |  |  |  |  |  |  |  |
| *Drepanocephalus cf. spathans HP1010.1 bc1* | *Nannopterum auritus* | USA: New York | - | - | - | - | PZ885078 | - | This Study |
| *Drepanocephalus cf. spathans HP1010.1 bc 2* | *Nannopterum auritus* | USA: New York | - | - | - | - | - | PZ885721 | This Study |
| *Drepanocephalus cf. spathans HP1010.1 bc 3* | *Nannopterum auritus* | USA: New York | - | - | - | - | PZ885079 | - | This Study |
| *Drepanocephalus cf. spathans HP1010.1 bc 4* | *Nannopterum auritus* | USA: New York | - | - | - | - | PZ885080 | - | This Study |
| *Drepanocephalus cf. spathans HP1010.1 bc6* | *Nannopterum auritus* | USA: New York | - | - | - | - | - | PZ885724 | This Study |
| *Drepanocephalus cf. spathans HP1183.1A* | *Nannopterum auritus* | USA: New York | Hap_26 | 1 | PRJNA1518865 | PRJNA1518865 | PZ885083 | PZ885727 | This Study |
| *Drepanocephalus cf. spathans HP1183.1B* | *Nannopterum auritus* | USA: New York | Hap_25 | 1 | PRJNA1518865 | PRJNA1518865 | PZ885084 | PZ885728 | This Study |
| *Drepanocephalus cf. spathans HP1189.2A* | *Nannopterum auritus* | USA: New York | Hap_18 | 2 | PRJNA1518865 | PRJNA1518865 | PZ885085 | PZ885726 | This Study |
| *Drepanocephalus cf. spathans HP1189.2 bc 12* | *Nannopterum auritus* | USA: New York | - | - | - | - | PZ885082 | PZ885725 | This Study |
| *Drepanocephalus cf. spathans HP1189.2 bc13* | *Nannopterum auritus* | USA: New York | - | - | - | - | - | PZ885722 | This Study |
| *Drepanocephalus cf. spathans HP1189.2 bc14* | *Nannopterum auritus* | USA: New York | - | - | - | - | PZ885081 | PZ885723 | This Study |
|  |  |  |  |  |  |  |  |  |  |
| *Drepanocephalus mexicanus DNA2623* | *Nannopterum brasilianus* | Mexico | Hap_13 | 6 | KY636182 | - | MF351543 | KY636270 | Hernandez-Cruz et al 2017 |
| *Drepanocephalus mexicanus DNA2754* | *Nannopterum brasilianus* | Mexico | Hap_12 | 6 | KY636185 | - | - | KY636277 | Hernandez-Cruz et al 2017 |
| *Drepanocephalus mexicanus DNA2750* | *Nannopterum brasilianus* | Mexico | Hap_12 | 6 | KY636188 | - | - | KY636273 | Hernandez-Cruz et al 2017 |
| *Drepanocephalus mexicanus DNA1523* | *Nannopterum brasilianus* | Mexico | Hap_12 | 6 | KY636229 | - | - | KY636245 | Hernandez-Cruz et al 2017 |
| *Drepanocephalus mexicanus DNA2175* | *Nannopterum brasilianus* | Mexico | Hap_9 | 6 | KY636202 | - | - | KY636263 | Hernandez-Cruz et al 2017 |
| *Drepanocephalus mexicanus DNA1519* | *Nannopterum brasilianus* | Mexico | Hap_10 | 6 | KY636225 | - | - | KY636241 | Hernandez-Cruz et al 2017 |
| *Drepanocephalus mexicanus DNA1520* | *Nannopterum brasilianus* | Mexico | Hap_10 | 6 | KY636226 | - | - | KY636242 | Hernandez-Cruz et al 2017 |
| *Drepanocephalus mexicanus DNA2326* | *Nannopterum brasilianus* | Mexico | Hap_11 | 6 | KY636196 | - | MF351542 | - | Hernandez-Cruz et al 2017 |
| *Drepanocephalus mexicanus DNA2749* | *Nannopterum brasilianus* | Mexico | Hap_12 | 6 | KY636183 | KY636295 | MF351544 | KY636272 | Hernandez-Cruz et al 2017 |
| *Drepanocephalus mexicanus DNA2753* | *Nannopterum brasilianus* | Mexico | Hap_12 | 6 | KY636186 | KY636298 | - | KY636276 | Hernandez-Cruz et al 2017 |
| *Drepanocephalus mexicanus DNA2752* | *Nannopterum brasilianus* | Mexico | - | - | - | KY636297 | - | KY636275 | Hernandez-Cruz et al 2017 |
| *Drepanocephalus mexicanus DNA2325* | *Nannopterum brasilianus* | Mexico | Hap_4 | 7 | KY636197 | - | - | KY636267 | Hernandez-Cruz et al 2017 |
| *Drepanocephalus mexicanus DNA1522* | *Nannopterum brasilianus* | Mexico | Hap_5 | 7 | KY636228 | KY636281 | - | KY636244 | Hernandez-Cruz et al 2017 |
| *Drepanocephalus mexicanus DNA2618* | *Nannopterum brasilianus* | Mexico | Hap_6 | 7 | KY636184 | - | - | - | Hernandez-Cruz et al 2017 |
| *Drepanocephalus mexicanus DNA1521* | *Nannopterum brasilianus* | Mexico | Hap_6 | 7 | KY636227 | KY636280 | - | KY636243 | Hernandez-Cruz et al 2017 |
| *Drepanocephalus mexicanus DNA2751* | *Nannopterum brasilianus* | Mexico | Hap_7 | 7 | KY636187 | KY636296 | - | KY636274 | Hernandez-Cruz et al 2017 |
| *Drepanocephalus mexicanus DNA2324* | *Nannopterum brasilianus* | Mexico | Hap_1 | 6 | KY636198 | KY636283 | - | KY636266 | Hernandez-Cruz et al 2017 |
| *Drepanocephalus mexicanus DNA2323* | *Nannopterum brasilianus* | Mexico | Hap_2 | 7 | KY636199 | KY636282 | - | KY636265 | Hernandez-Cruz et al 2017 |
| *Drepanocephalus mexicanus DNA2327* | *Nannopterum brasilianus* | Mexico | Hap_3 | 7 | KY636195 | - | - | - | Hernandez-Cruz et al 2017 |
| *Drepanocephalus mexicanus DNA2174* | *Nannopterum brasilianus* | Mexico | - | - | - | - | - | KY636262 | Hernandez-Cruz et al 2017 |
|  |  |  |  |  |  |  |  |  |  |
| *Drepanocephalus sp. MJG-2014 HAP-H3* | *Biomphalaria straminea* | Brazil | Hap_8 | 11 | KP053256 | KP053264 | KP053261 | KP053261 | Pinto et al. 2016 |
| *Drepanocephalus sp. Echinocercaria_22* | *Biomphalaria straminea* | Argentina | - | - | - | - | - | - | Fernández & Laidemitt 2026 |
|  |  |  |  |  |  |  |  |  |  |
| *Chaunocephalus ferox CF1* | *Ciconia boyciana* | China | - | - | PQ676277 | - | PQ676287 | - | Zhou et al 2024 |
| *Chaunocephalus ferox E5* | Wild Bird' | Egypt | - | - | - | - | - | PP660909 | Saad et al. 2024 |
| *Chaunocephalus ferox DFBG2* | *Ciconia boyciana* | China | - | - | PZ282475 | - | - | PZ282482 | Kuang et al. 2026 |
| *Chaunocephalus ferox DFBG4* | *Ciconia boyciana* | China | - | - | PZ282476 | - | - | PZ282483 | Kuang et al. 2026 |
| *Chaunocephalus ferox DFBG9* | *Ciconia boyciana* | China | - | - | PZ282477 | - | - | PZ282484 | Kuang et al. 2026 |
| *Chaunocephalus ferox DFBG10* | *Ciconia boyciana* | China | - | - | PZ282478 | - | - | PZ282485 | Kuang et al. 2026 |
|  |  |  |  |  |  |  |  |  |  |
| *Euparyphium capitaneum DNA2615* | *Anhinga anhinga* | Mexico |  | - | KY636235 |  |  | KY636279 | Hernandez-Cruz et al 2017 |
| *Euparyphium capitaneum DNA2614* | *Anhinga anhinga* | Mexico |  | - | KY636236 | - | - | KY636278 | Hernandez-Cruz et al 2017 |
|  |  |  |  |  |  |  |  |  |  |
| *Pegosomum asperum* | Ardea alba | Germany | - | - | - | - | KY945919 | KY945919 | Hirzmann et al. 2017 |
| *Pegosomum asperum* | Ardea alba | Russia | - | - | - | - | PP388212 | - | Vlasenkov 2024 |
| *Pegosomum asperum 3LF-2541* | *Egretta alba* | Czech Republic | - | - | - | KX097858 | - | - | Heneberg et al. 2016 |
| *Pegosomum asperum 3LF-2542* | *Egretta alba* | Czech Republic | - | - |  | KX097859 | - | - | Heneberg et al. 2016 |
| *Pegosomum saginatum* | *Ardea alba* | Germany | - | - | - | - | KY945918 | KY945918 | Hirzmann et al. 2017 |
|  |  |  |  |  |  |  |  |  |  |
| Paryphostomum radiatum KM7 | *Phalacrocorax carbo* | Hungary | - | - | - | - | - | KM973000 | Molnar et al. 2014 |
| Paryphostomum radiatum KM5 | *Phalacrocorax carbo* | Hungary | - | - | - | - | - | KM972998 | Molnar et al. 2014 |
| Petasiger exaeretus PA3 | Rutilus rutilus | Hungary | - | - | - | - | - | KY283996 | Cech et. al. 2017 |
| Petasiger exaeretus KM2 | *Phalacrocorax carbo* | Hungary | - | - | - | - | - | KY283997 | Cech et. al. 2017 |
| Petasiger exaeretus KM4 | *Phalacrocorax carbo* | Hungary | - | - | - | - | - | KY283998 | Cech et. al. 2017 |
| *Neopetasiger neocomense AK73A* | *Podiceps cristatus* | Czech Republic | - | - | - | JQ425591 | - | - | Georgieva et al. 2012 |
| *Neopetasiger islandicus MGC* | *Planorbula armigera* | Canada: Alberta | - | - | - | KT831342 | - | - | Gordy et al. 2015 |
| *Petasiger islandicus AK213* | *Gyraulus cf. laevis* | Iceland | - | - | - | JQ425588 | - | - | Georgieva et al. 2012 |
| *Petasiger islandicus S217* | *Gyraulus cf. laevis* | Iceland | - | - | - | JQ425590 | - | - | Georgieva et al. 2012 |
| Petasiger radiatus KM | *Biomphalaria sudanica* | Kenya | - | - | - | MN745946 | - | - | Outa et al. 2020 |
| Petasiger radiatus BUK89 | *Phalacrocorax carbo* | Hungary | - | - | - | - | - | PP188703 | Gyongy et al. 2024 |
| Petasiger radiatus P2BV1 | *Burnupia transvaalensis* | South Africa | - | - | - | - | - | PP738869 | Outa & Avenant-Oldewage, 2025 |
| *Petasiger phalacrocoracis FP1120* | *Tinca tinca* | Lithuania | - | - | - | PX549899 | - | - | Kudlai et al. 2025 |
| Petasiger phalacrocoracis FP1319 | *Cobitis taenia* | Lithuania | - | - | - | - | PX600353 | - | Kudlai et al. 2025 |
| Petasiger phalacrocoracis AK236 | *Phalacrocorax carbo* | Czech Republic | - | - | - | JQ425593 | - | - | Georgieva et al. 2012 |
| *Petasiger phalacrocoracis PP1* | *Phalacrocorax carbo* | Tanzania | - | - | MZ394506 | - | - | - | Chibwana & Katandukila 2021 |
| *Petasiger phalacrocoracis PP3* | *Phalacrocorax carbo* | Tanzania | - | - | MZ394508 | - | - | - | Chibwana & Katandukila 2021 |
| *Petasiger phalacrocoracis PP2* | *Phalacrocorax carbo* | Tanzania | - | - | MZ394509 | - | - | - | Chibwana & Katandukila 2021 |
| Petasiger phalacrocoracis 4Es001 | Ampullaceana balthica | Denmark | - | - | - | - | - | MW001052 | Duan et al. 2021 |
| Petasiger phalacrocoracis E0275 | Ampullaceana balthica | Germany | - | - | - | - | - | PZ225369 | Huesken 2026 |
| Petasiger phalacrocoracis E1235 | Ampullaceana balthica | Germany | - | - | - | - | - | PZ225376 | Huesken 2026 |
| *Petasiger sp. 1 MSB:Para:26651* | *Radix natalensis* | Kenya | - | - | - | MK534361 | - | - | Laidemitt et al 2019 |
| *Petasiger sp. 3 MSB:Para:26654* | *Radix natalensis* | Kenya | - | - |  | MK534364 | - | - | Laidemitt et al 2019 |
| *Petasiger sp. 1 GAHN1* | *Gyraulus albus* | Germany | - | - | - | KM191808 | - | - | Selbach et al. 2014 |
| *Petasiger sp. 2 MSB:Para:26657* | *Bulinus globosus* | Kenya | - | - | - | MK534366 | - | - | Laidemitt et al 2019 |
| *Petasiger sp. 2 MSB:Para:26660* | *Bulinus globosus* | Kenya | - | - | - | MK534369 | - | - | Laidemitt et al 2019 |
| *Petasiger sp. 3 GAHN6* | *Gyraulus albus* | Germany | - | - | - | KM191812 | - | - | Selbach et al. 2014 |
| *Petasiger sp. 4 GAG* | *Gasterosteus aculeatus* | Canada: | - | - | - | KM191817 | - | - | Selbach et al. 2014 |
| *Petasiger sp. 4 MSB:Para:26632* | *Biomphalaria pfeifferi* | Kenya | - | - | - | MK534350 | - | - | Laidemitt et al 2019 |
| *Petasiger sp. 5 BMC897* | *Planorbella trivolvis* | Canada: Alberta | - | - | - | PV595381 | - | - | McPhail et al. 2025 |
| *Petasiger sp. 5 MDBu13* | *Bulinus sp.* | South Africa | - | - | PX775916 | - | - | - | Hüsken et al. 2026 |
| *Petasiger sp. 5 BOBu30* | *Bulinus sp.* | South Africa | - | - | PX775912 | - | - | - | Hüsken et al. 2026 |
| *Petasiger sp. 5 MSB:Para:26644* | *Bulinus sp.* | Kenya | - | - | - | MK534355 | - | - | Laidemitt et al 2019 |
| *Petasiger sp. 5 MSB:Para:26688* | *Bulinus truncatus trigonus* | Kenya | - | - | - | MK534396 | - | - | Laidemitt et al 2019 |
| Petasiger sp. ER1P20LH | *Pseusosuccinea columella* | South Africa | - | - | - | - | - | PP228840 | Outa et al. 2024 |
| Petasiger sp. R6P24P | *Pseusosuccinea columella* | South Africa | - | - | - | - | - | PP228841 | Outa et al. 2024 |
| Petasiger sp. P4BV1 | *Burnupia transvaalensis* | South Africa | - | - | - | - | - | PP738870 | Outa & Avenant-Oldewage, 2025 |
| *Petasiger sp. P6BV1* | *Burnupia transvaalensis* | South Africa | - | - | PP738976 | - | - | - | Outa & Avenant-Oldewage, 2025 |
| *Petasiger sp. P7BV1* | *Burnupia transvaalensis* | South Africa | - | - | PP738977 | - | - | - | Outa & Avenant-Oldewage, 2025 |
|  |  |  |  |  |  |  |  |  |  |
| *Echinostoma miyagawai* | *Anas platyrhynchos* | Ukarine | - | - | - | - | KT956916 | - | Tkach et al. 2015 |
| *Echinostoma trivolvis Worm23* | *Ondatra zibethicus* | USA: Virgina | - | - | - | JQ670861 | - | - | Detwiler et al. 2012 |
| *Echinostoma trivolvis* | *Planorbella pilsbryi* | USA: Wisconsin | - | - | - | PQ421228 | PQ459505 | - | Schumacher and Minchella 2024 |
| *Echinostoma trivolvis JP2023HtE5* | *Planorbella trivolvis* | USA: Indiana | - | - | - | PQ421239 | - | - | Schumacher and Minchella 2024 |
| *Echinostoma trivolvis JP2023HtE4* | *Planorbella trivolvis* | USA: Indiana | - | - | - | PQ421242 | - | - | Schumacher and Minchella 2024 |
| *Echinostoma bolschewense EBG7* | *Viviparus acerosus* | Slovakia | - | - | - | KP065614 | - | - | Georgieva et al. 2014 |
| Echinostomatidae sp. ER1BV2a | *Burnupia mooiensis* | South Africa | - | - | - | - | - | PP738871 | Outa & Avenant-Oldewage, 2025 |
